# Insight into the physiological and pathological roles of the aryl hydrocarbon receptor pathway in glucose homeostasis, insulin resistance, and diabetes development

**DOI:** 10.1186/s11658-022-00397-7

**Published:** 2022-11-22

**Authors:** Tahseen S. Sayed, Zaid H. Maayah, Heba A. Zeidan, Abdelali Agouni, Hesham M. Korashy

**Affiliations:** 1 Department of Pharmaceutical Sciences, College of Pharmacy, QU Health, Qatar University, 2713, Doha, Qatar; 2 American School of Doha, Doha, Qatar

**Keywords:** Aryl hydrocarbon receptor, Diabetes mellitus, Glucose hemostasis, Insulin, Environmental toxicants

## Abstract

The aryl hydrocarbon receptor (AhR) is a ligand-activated transcriptional factor that mediates the toxicities of several environmental pollutants. Decades of research have been carried out to understand the role of AhR as a novel mechanism for disease development. Its involvement in the pathogenesis of cancer, cardiovascular diseases, rheumatoid arthritis, and systemic lupus erythematosus have long been known. One of the current hot research topics is investigating the role of AhR activation by environmental pollutants on glucose homeostasis and insulin secretion, and hence the pathogenesis of diabetes mellitus. To date, epidemiological studies have suggested that persistent exposure to environmental contaminants such as dioxins, with subsequent AhR activation increases the risk of specific comorbidities such as obesity and diabetes. The importance of AhR signaling in various molecular pathways highlights that the role of this receptor is far beyond just xenobiotic metabolism. The present review aims at providing significant insight into the physiological and pathological role of AhR and its regulated enzymes, such as cytochrome P450 1A1 (CYP1A1) and CYP1B1 in both types of diabetes. It also provides a comprehensive summary of the current findings of recent research studies investigating the role of the AhR/CYP1A1 pathway in insulin secretion and glucose hemostasis in the pancreas, liver, and adipose tissues. This review further highlights the molecular mechanisms involved, such as gluconeogenesis, hypoxia-inducible factor (HIF), oxidative stress, and inflammation.

## Introduction

### Diabetes mellitus

Diabetes mellitus (DM) is a chronic metabolic disease characterized by elevated blood glucose levels resulting from impaired insulin secretion/action and glucose homeostasis [1]. The global prevalence of DM has markedly increased during the last few decades, from 4.7% in 1980 to about 10.5% in 2020, with the expectation to increase to 10% in 2035 [1, 2]. The World Health Organization (WHO) estimated DM as the seventh leading cause of death in 2016, and the global health spending on DM in 2013 reached approximately USD 548 billion, and is expected to increase to USD 627 billion by 2035 [1]. There are two main types of DM depending on the insulin secretory function of the pancreas: type 1 DM (T1DM), which is characterized by a complete lack of insulin secretion and frequently occurs in children and adolescents, and type 2 DM (T2DM), which frequently occurs in adults and results mainly from reduced insulin secretion and function [3]. T2DM is the most common, and accounts for more than 90% of all DM cases worldwide. Although the condition also develops with age, if left untreated it can lead to damage to other vital tissue systems. Changes in lipid metabolism, blood pressure, and the onset of obesity are the most frequent parameters indicative of the development of T2DM [3]. Although the factors contributing to the development of T2DM are well identified and characterized, these traditional risk factors alone cannot explain the rapidly increasing prevalence of diabetes worldwide. Thus, other factors beyond these conventional risk factors, such as exposure to environmental pollutants, are now being studied. This review highlights the impact of exposure to environmental toxins and the activation of the transcriptional factor, aryl hydrocarbon receptor (AhR), on glucose hemostasis, insulin secretion, and DM development.

### Environmental pollution and AhR

**Fig. 1:**
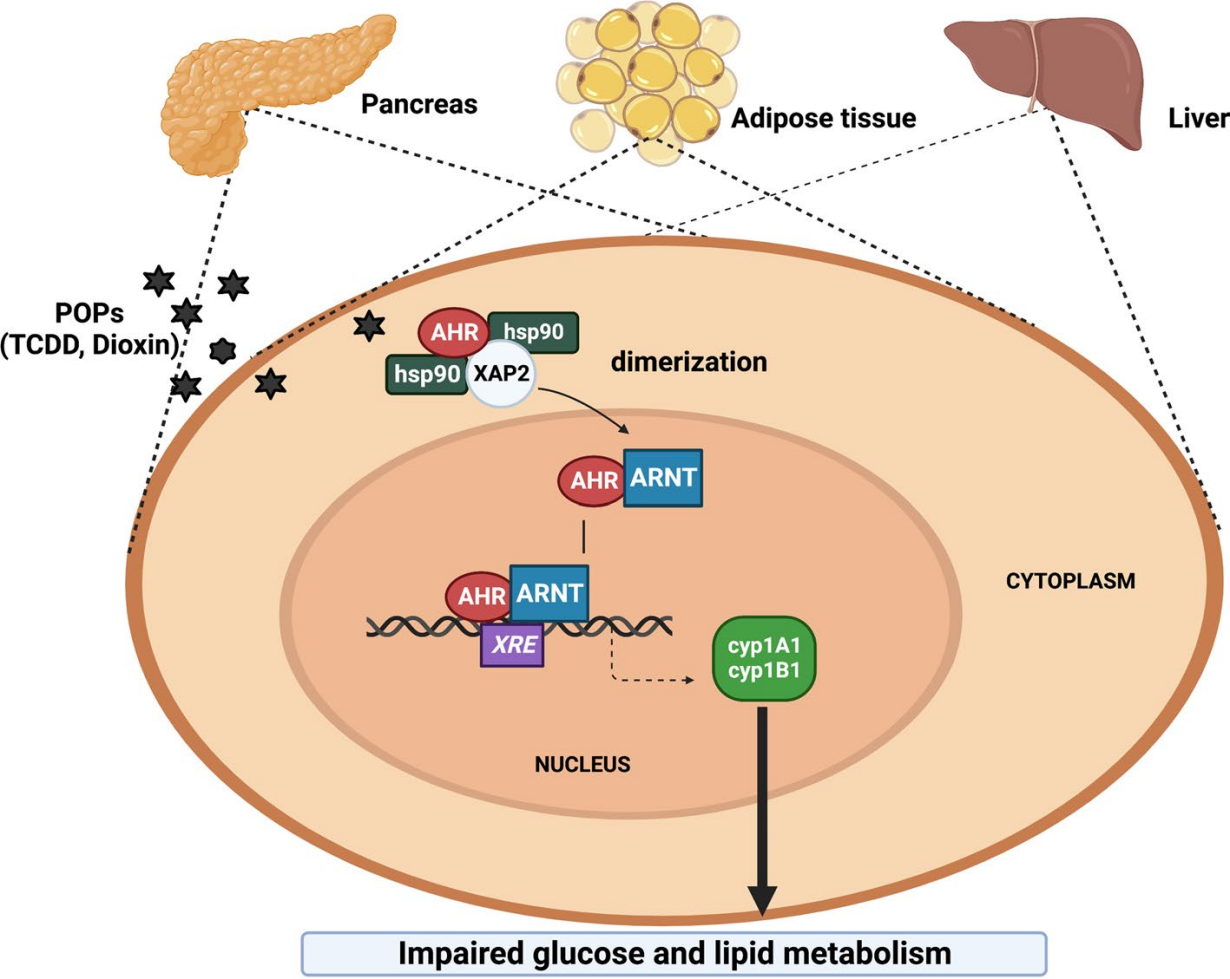
The proposed pathway for AhR-mediated glucose hemostasis and DM by environmental pollutants. AhR is expressed in various tissues, including the pancreas, liver, and adipose tissues, making it a crucial receptor in many physiological and pathological processes involved in insulin secretion and glucose metabolism. Mechanistically, upon binding of TCDD to AhR, the activated receptor translocates to the nucleus after dissociation from its inhibitory protein HSP90 and XAP2. The activated receptor interacts with type 2 basic helix–loop–helix/PER-ARNT-SIM (bHLH-PAS) protein, AhR nuclear translocator (ARNT). The resulting complex selectively binds to a specific sequence on CYP1A1 DNA. known as the xenobiotic responsive element (XRE), leading to transcriptional induction of specific genes involved in xenobiotic metabolism, such as CYP1A1 and CYP1B1. Induction of AhR downstream targets CYP1A1 and CYP1B1 directly or indirectly induce impaired glucose hemostasis and insulin secretion. Created by bioRender.com

The development of human activities has resulted in the production and release of numerous chemicals into the air, water, and soil, causing massive environmental pollution [4]. Chronic exposure of humans to a mixture of environmental chemicals and pollutants, such as dioxins and other polycyclic aromatic hydrocarbons (PAHs), causes organ toxicity through the activation of a cytosolic transcription factor known as the Aryl hydrocarbon receptor (AhR). AhR regulates cell differentiation, proliferation, and cancer imitation [5–7]. Activation of AhR upon binding to its ligand, such as 2,3,7,8-tetrachlorodibenzo[p]dioxin (TCDD) or 7,12-dimethybenz[a]anthracene (DMBA), induces the transcription of a group of xenobiotic metabolizing enzymes, cytochrome P4501A1 (CYP1A1), CYP1B1, and CYP1A2 [8, 9]. Mechanistically, the binding of environmental pollutants to the AhR activates its translocation to the nucleus after dissociation from its inhibitory protein, heat shock protein (HSP90). In the nucleus, the activated receptor interacts with a transcription factor, AhR nuclear translocator (ARNT), and the resulting complex then selectively binds with a specific sequence on CYP1A1 DNA, known as the xenobiotic responsive element (XRE) to start the transcription process of CYP1A1 and CYP1B1 (Fig. 1) [10, 11].

### Environmental pollution and DM

The available information suggests that the concentrations of chemicals and toxins in the environment, even in trace amounts, can cause severe problems for all organisms, including humans [12]. Environmental pollutants such as heavy metals and PAHs, are among the most hazardous and toxic [13]. The tissue accumulation of toxins from contaminated air, water, soil, and food plays a vital role in the pathogenesis of diseases. A systematic review has provided convincing evidence supporting the role of environmental pollutants in DM development [14]. Recent studies have reported that exposure to airborne fine particular matter 2.5 (PM2.5), increases the prevalence of T2DM and the glycosylated hemoglobin (HbA1c) levels among both diabetic and healthy subjects [15, 16], and significantly decreases insulin sensitivity in obese participants [17]. A recent case-cohort study in Brazil involving 1605 non-diabetic participants with 4 years of follow-up, revealed that those exposed to persistent organic pollutants (POPs) with more than a twofold increase in the AhR ligand levels, demonstrated a higher risk of developing diabetes [18].

In recent years, epidemiological studies have shown that elevated serum levels of several POPs are linked to metabolic syndromes including obesity [19], diabetes [20], hypertension [21], and inflammatory diseases [22]. AhR agonists, including beta naphthoflavone (β-NF), have been reported to suppress preadipocyte differentiation, downregulate glucose transporting activities, modulate inflammatory responses, and interfere with estrogen in cultured cell and animal models. Studies have also reported a link between exposure to organochlorines and disrupted blood-glucose regulation and diabetes. In addition, Epidemiological studies on industrial workers and population groups in high-exposure environments have linked higher polychlorinated biphenyl (PCB) body burden to increased risk of altered glucose metabolism and diabetes [23]. This was previously supported by the observations of Longnecker et al. who reported that diabetic pregnant women exhibited higher PCB levels than non-diabetic women [24].

Similarly, other studies show dose-dependent relationships between diabetes or fasting-glucose levels and PCBs [25, 26]. A cross-sectional study by Codru et al., found a significant association between serum PCB and pesticide levels and diabetes in adult Native-American population after adjustment for age, body mass index (BMI), serum lipid levels, sex, and smoking [27]. Although the study lacks data that highlight the exact cause and effect of this observed pattern, there is emerging evidence that environmental exposure to persistent organochlorine compounds is associated with an elevated incidence of diabetes [27].

All these studies strongly support the hypothesis that exposure to environmental pollutants contributes to DM development. However, there are no reviews exploring the molecular mechanisms of the effect of AhR/CYP1A1 pathway modulations following exposure to environmental pollutants on glucose homeostasis and DM development.

## The role of AHR/CYP1A1 pathway in glucose homeostasis and insulin secretion

### Physiological role of AhR/CYP1A1 in glucose homeostasis and insulin secretion

The relative importance of AhR signaling within numerous molecular pathways is governed by evidence that points toward the role of the receptor beyond xenobiotic metabolism and detoxification. Several AhR knockout models have revealed many developmental defects, the most prominent being disruption of hepatic functioning [28] and other physiological processes such as cardiovascular physiology [29]. More specifically, it was reported that inactivation of the basal activity of the AhR/CYP pathway either genetically or chemically leads to impairment of the heart, liver, spleen, and skin tissues [30, 31]. AhR is a crucial receptor required by a multitude of physiological processes. However, there is a lack of information on the physiological role of AhR in DM [32]. To better understand the physiological role of the AhR/CYP1A1 pathway in glucose homeostasis and DM pathogenesis, it is essential to first explore the localization and expression of the AhR and its dependent genes in the glucose-regulated organs. This section reviews the most recent studies on the expression, function, and tissue localization of AhR and mediated CYP genes in the pancreas, liver, and adipose tissues.

AhR is one of the best-characterized bHLH/PAS proteins, expressed in various tissues among the mammalian and non-mammalian vertebrate species [33–35]. Studies in rodents have shown predominant receptor localization in the lung, thymus, kidney, and liver, and reduced expression in the heart and spleen [36]. A study comparing the mRNA expression of AhR in normal pancreas and chronic pancreatitis demonstrated a 2.8-fold higher AhR expression in the acinar and ductal cells of chronic pancreatitis tissues than in normal pancreas [37]. On the other hand, Gunton et al., highlighted the reduced levels of ARNT expression within pancreatic islets [38]. Furthermore, it has been found that expression of AhR/CYP1A1 within pancreatic β-cells may facilitate production of reactive intermediates [39]. Of particular interest are the CYP1A family proteins, which show significant expression in the pancreas itself [40, 41]. Several in vivo studies showed that pancreatic islets exposed to TCDD, a potent AhR/CYP1A1 inducer, reduced insulin secretion [42–45]. Recently, Ibrahim and coworkers reported that transient exposure of human and mouse pancreatic endocrine cell lines to TCDD causes significant suppression of insulin secretion, lowers plasma insulin levels, and increases β-cell death [46]. These effects were accompanied by an upregulation in the pancreatic function of the CYP1A1 enzyme, which were diminished in *cyp1a1* and *cyp1a2* knockout mouse islets [46].

Although AhR was initially identified due to its role in TCDD-induced toxicity, AhR knockout in vivo mouse models have demonstrated the role of the receptor in normal development and physiology. Exposure to TCDD, with subsequent activation of AhR, disrupts insulin secretion and glucose homeostasis in an AhR-dependent manner [47]. For example, Thackaberry et al. and others, reported imbalanced glucose homeostasis, decreased plasma levels of insulin, and impaired glucose intolerance in AhR knockout mice [47, 48], suggesting the importance of AhR expression in the maintenance of glucose and lipid homeostasis, as well as highlighting that deficiency of AhR has detrimental metabolic effects.

Gestational diabetes is marked by glucose intolerance and insulin resistance [49]. The onset of gestational diabetes has been shown to increase neonatal body weight, influenced by the maternal genotype alone [50]. On the contrary, Thackaberry et al. demonstrated that pregnant mice lacking AhR exhibit decreased fasting plasma insulin levels and insulin resistance, while hyperglycemia and altered glucose tolerance were not observed in these mice [47]. The onset of mature-onset diabetes of the young-2 (MODY-2), a type of gestational diabetes, is due to the disruption of a single copy of the glucokinase gene, causing alterations in the sensing of glucose and decreased insulin secretion [47]. An interesting point to note is that although MODY-2 shares similarities with the AhR-null mice phenotype of pregnant mice, MODY-2 mice develop mild hyperglycemia under fasting conditions and glucose intolerance, but do not develop insulin resistance, unlike AhR-null mice [47]. These studies suggest that as AhR-null female mice age, they develop altered insulin regulation, indicating that AhR is vital in regulating insulin in pregnant and non-pregnant female mice.

### Pathological role of the AhR/CYP1A1 pathway in T1DM development

Exposure of young children to environmental pollutants has been linked to the accelerated manifestation of T1DM [51]. AhR is widely expressed in many innate immune and anti-inflammatory cells [52], which has led to studies deciphering the possible role of AhR in the pathogenesis of T1DM. Emerging data have put forward the role of AhR signaling in rectifying β-cell destruction caused by disrupted immune homeostasis. The complex formation of AhR with many transcriptional factors, controls the expression of critical genes necessary for autoimmune responses during T1DM development. For instance, AhR activation destroys β-cells through suppression of effector T cell function via direct targeting of the effector T cell subsets or indirect induction of regulatory T cells (Treg cells) [53]. Consequently, interferon-γ (IFN-γ) and tumor necrosis factor-α (TNF-α) are major molecules leading to β-cell death, and T cells deficient in AhR produce more IFN-γ [54]. In addition, Maltepe et al. showed that genes that respond to hypoxia and hypoglycemia are not activated in ARNT-deficient embryonic stem cells [55].

A review by Tiantian et al. provides substantial evidence for the potential involvement of AhR in T1DM pathogenesis [56], which has been implicated as AhR is widely expressed in immune cells. AhR signaling has also been found to modulate autoimmune responses during T1DM development. AhR interacts with a multitude of immune cells, such as antigen-presenting cells (dendritic cells and macrophages), gut innate immune cells (Innate Lymphoid cells (ILCs), Intraepithelial lymphocytes (IELs), and T cells), and adaptive immune cells (Tregs, Tr1, Th1, and Th17 cells) [56]. Some vital immune cells, such as the dendritic cells, play a critical role in initiating autoimmune responses against pancreatic cells. Moreover, these cells are one of the earliest islets infiltrating leukocytes that are also essential in activating lymphocytes in the early insulitis stage [57].

Furthermore, a recent study by Miani et al. in non-obese diabetic (NOD) mice [58], shed light on the complex interaction between gut microbiota, immune cells, and pancreas in the development of T1DM [58]. NOD mice have been extensively used as a prime animal model for studying autoimmune diabetes [59]. The study highlights the plausible association of pancreatic endocrine cells in crosstalk with gut microbiota metabolites in NOD mice through the secretion of antimicrobial peptides (AMPs) that exert an immune-regulatory function to halt pancreatic inflammation [58]. In addition, AhR is notably expressed in intestinal ILCs and is mainly involved in driving the development of gut ILC22 cells, therefore pushing toward antimicrobial function and mucosal epithelial cell survival and proliferation [60, 61]. Taken together, these data highlight that an AhR–ILCs axis is essential to maintain gut integrity, which in turn impacts T1DM progression via crosstalk between the gut and pancreas.

In healthy individuals, pathogenic autoimmunity is controlled by a specialized subset of T cells named Treg cells [62]. These cells undergo differentiation and their function depends on the forkhead box P3 (Foxp3) transcription factor [63, 64]. Foxp3 is an essential factor, therefore gene mutations could lead to immune dysregulation. Additionally, Treg cell development is also associated with Interleukin 17 (IL-17)-producing T-cells (Th17) and transforming growth factor beta (TGF-β1), which induces differentiation of these cells [65]. Based on this, one study reported that AhR activation regulates the generation of Treg and Th17 cells in C57BL/6 mice injected with TCDD. The study further established that TCDD, a potent AhR activator, directly binds to the Foxp3 promoter, leading to Treg induction and development [66], suggesting there is a link between Foxp3 and Tregs type 1 regulatory T cells (Tr1 cells), and AhR [53, 67]. These studies demonstrate that AhR is a molecular sensor for external and internal signaling, employing numerous exogenous and endogenous ligands. These properties render it a viable therapeutic target, paving the way toward developing AhR-targeted immunomodulatory agents.

### Pathological roles of AhR/CYP1A1 pathway in T2DM development

The onset of T2DM is characterized by the disruption of the insulin secretion pathway in the islets of Langerhans, caused by mutant genes that result in β-cell dysfunction [68] and insulin resistance [69]. A better understanding of the mechanism requires studying the molecular basis of pancreatic islets. Kubi et al. have shown that treatment of human embryonic stem cells (hESCs) with low doses of TCDD, an AhR activator, caused impairment of the differentiation of the pancreatic lineage and AMP-activated protein kinase (AMPK) pathway, leading to impaired pancreatic development and function [70]. In addition, it has been reported that induction of experimental diabetes in rats using streptozotocin (STZ) was associated with elevated CYP1A1 activity levels in diabetic rats compared with the control [71]. A study by Dabir et al. reported activation of AhR expression and function within aortic endothelial cells (ECs) in response to elevated glucose levels. This activation of AhR induces the expression of thrombospondin-1 gene promoter (TSP-1), a potent antiangiogenic and proatherogenic protein that plays a role in the development of diabetic vascular complications [72]. It has been postulated that under hyperglycemic conditions, AhR complexes with specific transcriptional factors, early growth response (EGR-1), and activator protein-2 (AP-2), that mediate changes in ECs gene expression, leading to endothelial dysfunction and other vascular diseases [72]. Adequate glycemic control in diabetes is a key factor in managing the metabolic condition to prevent the onset of other microvascular complications [73]. An association between diabetes complications and AhR has been reported. For example, it has been demonstrated that patients with diabetic nephropathy showed elevated levels of serum AhR [74], which is further correlated to the increased levels of reactive oxygen species (ROS) and DNA damage, indicating the potential role of AhR in the pathogenesis of diabetic nephropathy [75]. Furthermore, Park et al. showed that sera of Korean diabetic and prediabetic patients express higher AhR ligand TCDD levels than non-diabetics, which were positively associated with obesity, blood pressure, and serum triglyceride, and fasting glucose levels. This was further supported in vitro, where co‐culturing of mouse myoblast C2C12 cells with sera of diabetic patients resulted in reduced intracellular ATP levels and elevated ROS generation compared with controls [76].

A linear correlation between serum AhR ligand activity and BMI in healthy and glucose-intolerant patients reveals that AhR ligands most likely play a role in body mass regulation. In patients with pancreatic cancer, a direct correlation between the plasma level of AhR with age and BMI was observed, specifically in pancreatic cancer patients > 65 years old and BMI < 25 kg/m^2^ [77, 78]. Since age is a crucial factor in the development of diabetes [79], it has been suggested that age and BMI could be significant contributing factors to elevated AhR levels. Moreover, the findings of Park et al. and others that sera from glucose intolerant subjects inhibit the mitochondrial functioning of cultured cells, further support the possibility that circulating AhR ligands contribute to mitochondrial dysfunction in tissues and eventually leading to insulin resistance and T2DM development [76, 80]. Taken together, findings from these studies suggest that activation of AhR/CYP1A1 in pancreatic islets following exposure to environmental toxins, such as TCDD, regulates glucose homeostasis and insulin secretion.

#### Pathological role of AhR/CYP1A1 in the pancreas

**Fig. 2:**
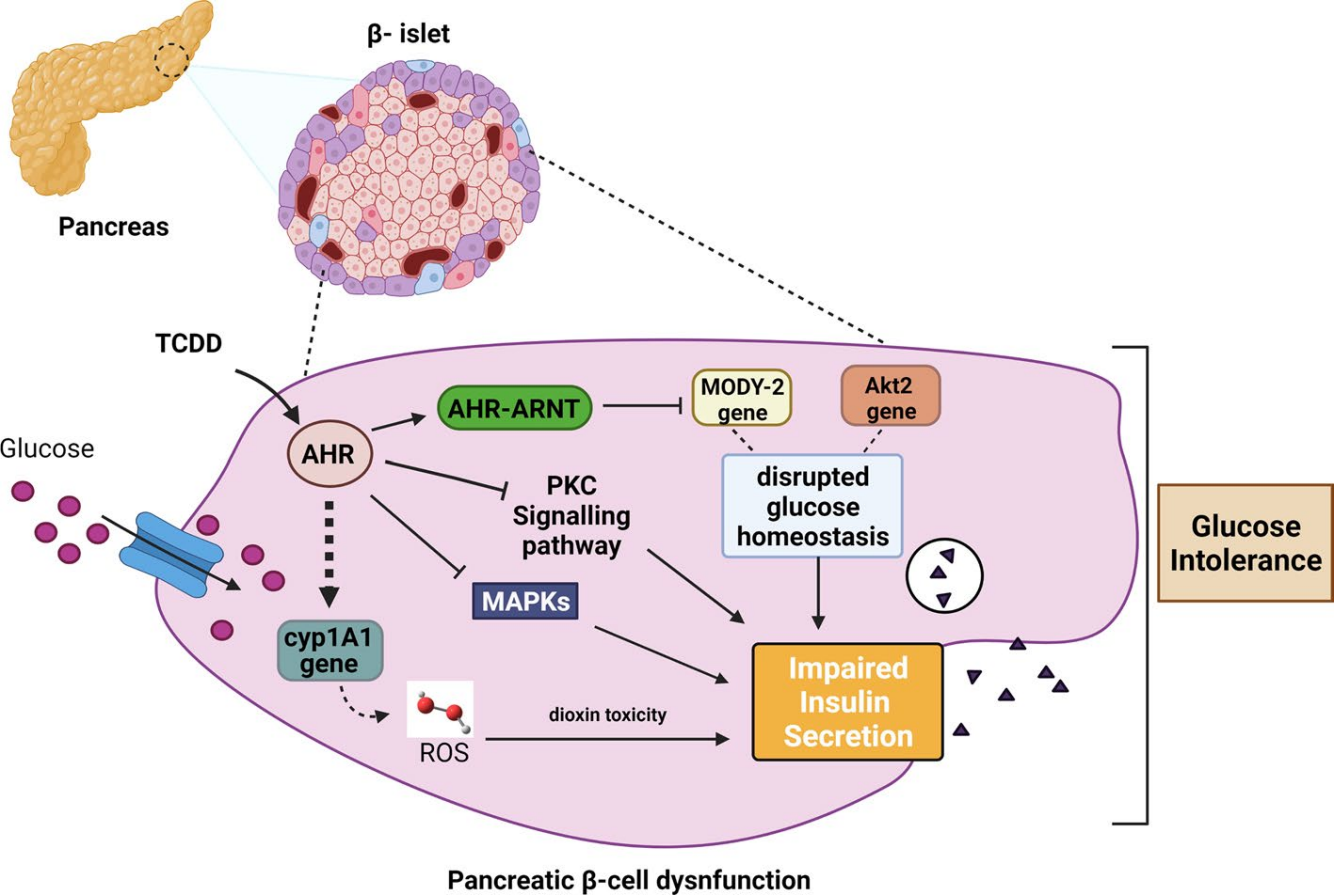
Pathological role of AhR/CYP1A1 in the pancreas: activation of AhR in the pancreatic islets upon exposure to ligands such as TCDD, leads to induction of CYP1A1, with subsequent generation of ROS and disruption of PKC and mitogen activated protein kinases (MAPK) signaling pathways, resulting in impaired insulin secretion and glucose intolerance within the islet. In addition, AhR–ARNT complexes formed in the pancreas also regulate the expression of specific genes, such as *MODY-2* and *Akt-2*, which are critical to glucose metabolism and hemostasis. Created by bioRender.com

Mounting evidence suggests the potential role of environmental chemicals in causing β-cell injury in the endocrine pancreas and further disrupting glucose homeostasis [42, 45, 47]. A previous study in rodents demonstrated that transient exposure to TCDD induced long-term activation of the CYP1A1 enzyme within pancreatic islets compared with the liver, despite a relatively small amount of TCDD reaching the pancreas [46], suggesting a possible involvement of CYP1A1 in insulin secretion by β-cells. Further studies have investigated the pancreatic toxicity of TCDD under in vivo, ex vivo, and in vitro experiments. These studies showed that TCDD disrupted the second-phase insulin secretion from islets in an AhR-dependent manner [45, 81]. The possibility that protein kinase C (PKC) pathway to regulate the second phase of insulin secretion is supported by the fact that TCDD enables the activation of PKC by translocating it from the cytoplasm to the cellular membrane [82]. Additionally, it is important to note that the ARNT gene is located on human chromosome 1q21. This region has replicated linkage to T2DM in diverse populations, indicating that altered insulin secretion could be due to specific ARNT variants [83]. This suggests that TCDD-induced insulin secretion impairment is indirectly caused by disruption of the PKC signaling pathway (Fig. 2).

Contrarily, studies have reported the activation of AhR as a consequence of high glucose levels. It has been shown that the AhR–XRE complex in the nuclear extract of endothelial cells is stimulated by high glucose. Furthermore, hyperglycemia has been found to affect the expression of endothelial proteins such as TSP-1 [84]. The link between TSP-1 and AhR has been further studied by Dabir et al. who report that high glucose level rapidly activates AhR in endothelial cells, and that AhR further controls the expression of the TSP-1 protein [72], triggering endothelial dysfunction and vascular disease. However, more studies are needed to explore further the effect of hyperglycemia on the AhR/CYP1A1 pathway.

#### Pathological role of AhR/CYP1A1 in the liver

Several studies have also shown the direct and indirect modulation of several CYP enzymes under diabetic conditions. Studies on AhR activation are associated mainly with regulating and stimulating CYP1A1, CYP1A2, and CYP1B1 in hepatocytes in response to compounds such as dioxins, dibenzofurans, and PCBs, reviewed in [85–87]. This was investigated using diabetic rats as animal models to decipher the regulatory influence of endocrine hormones on various organ systems. Imbalanced insulin levels have been found to modulate hepatic CYP1A2, CYP2A1, CYP2B1, CYP2C7, CYP3A1, and CYP3A2 in the hepatic microsomes of rats. Previous studies have shown that a DM-induced increase in hepatic ethoxyresorufin O-deethylase (EROD) activity was linked to a rise in CYP1A2 protein [88]. This was further supported by investigations demonstrating that increased CY1A2 and CYP2B activities are attributed to hyperketonemia that characterizes diabetes [89].

Expression of AhR and ARNT in normal developing tissues, as well as within embryonic tissues has raised speculation regarding their potential to disrupt development. A study showed the spatial and temporal expression pattern of AhR in C57BL/6N embryos from gestation day (GD) 10 through GD16 within various tissues. Increasing nuclear localization of AhR within hepatocytes was observed with advancing developmental age, demonstrating uniform expression levels of the receptor across the liver, and highlighting the role of this receptor in normal embryonic development [90]. In another study, liver mRNA profiles of wild-type and AhR-null mice showed higher levels of TGF-β in the liver of AhR-null mice, which contributed to fibrosis development and reduced retinoic acid metabolism [91, 92]. Evidence shows that retinoic acid signaling is required in adult mice pancreas for maintaining both β-cell function and mass [93], indicating that the AhR is relevant to preserving normal cell physiology.

**Fig. 3:**
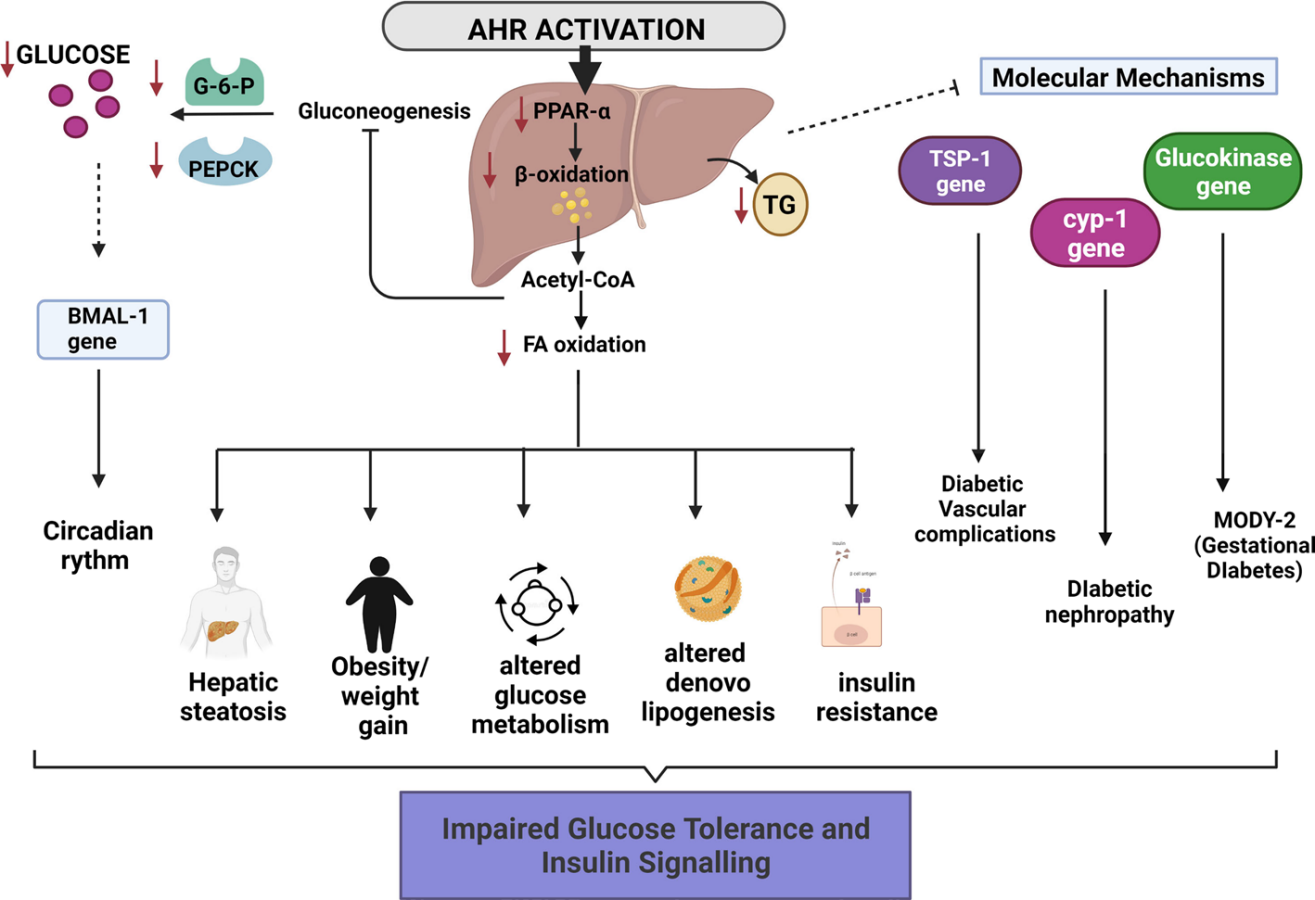
Pathological role of AhR/CYP1A1 in the liver: AhR activation effects on the liver and subsequent impact on the development of T2DM. Activation of AhR in the liver initiates model crosstalk between AhR, PPAR-α signaling, circadian clock dysfunction, and several key regulatory pathways. AhR activation results in a decrease in PPAR-α levels, which further impacts β-oxidation. This is accompanied by decreased expression levels of PEPCK and G6Pase, known for regulating hyperglycemia and insulin resistance. Moreover, since PPAR-α also exhibits circadian variation, it influences CLOCK and BMAL1 levels, altering glucose tolerance and disrupting the regulation of specific metabolism genes. AhR has also been shown to control the expression of the TSP-1 protein, an antiangiogenic and proatherogenic protein involved in development of several vascular diabetic complications. Since many molecular pathways are regulated by AhR activation in the liver, these effects may implicate a subsequent impact on the development of T2DM. Created by bioRender.com

Data from previous studies have also revealed a complex interaction between AhR/ARNT and circadian proteins, such as circadian locomotor output cycles kaput (CLOCK) and brain, muscle ARNT-like protein 1 (BMAL1), also known as ARNT-like (ARNTL) owing to structural similarities between them. The activation of AhR causes the disruptive functioning of CLOCK/BAML1 in many tissues [94], which is known to alter glucose tolerance and essential metabolism genes [94–96]. Another study highlighted the association between the AhR and the peroxisome proliferator-activated receptor (PPAR-α), which is known to play a role in regulating glucose and lipid homeostasis [97], shedding light on glucose metabolism, insulin sensitivity, and circadian rhythm in the liver [98]. PPAR activation is known to increase the phosphoenolpyruvate carboxykinase (PEPCK) and glucose-6-phosphatase (G6Pase) expression, causing hyperglycemia and insulin resistance [99]; therefore, reduced expression of this receptor protein could confer a mechanism of protection against insulin resistance, reducing the risk of diabetes and its complications [100]. Importantly, AhR knockout mice models exhibited enhanced insulin sensitivity, improved glucose tolerance, and lower expression levels of PPAR [100]. Similarly, earlier studies have implicated the influence of PPAR-α signaling on AhR activation and circadian clock dysfunction leading to the onset of T2DM with insulin resistance in humans exposed to environmental toxins [99–101] (Fig. 3).﻿

One study investigated the effect of AhR agonists β-NF and TCDD on PPAR-α expression and glycolysis in murine hepatoma cell lines, Hepa1c1c7, and in AhR knockout mice. In the treatment of AhR knockout mice with AhR agonists, enhanced insulin sensitivity decreased the expression of PPAR-α. In addition, they observed an altered circadian rhythm of liver genes controlling glucose and fatty acid metabolism, such as G6Pase and PEPCK. In the in vitro Hepa1c1c7 cells, TCDD, and β-NF induced Cyp1a1 and PPAR-α expression [102]. These results were further validated by silencing Bmal1 in Hepa1c1c7 cell line, which resulted in decreased expression of PPAR, AhR, and Cyp1a1. Furthermore, AhR silencing inhibited Bmal1, whereas Ahr and Bmal1 siRNA reduced PPAR-α and glucose metabolism genes. Since PPAR-α regulates BMAL1, AhR, and CYP1A1, PPAR-α may mediate the inhibitory effect of Bmal1 silencing on the AhR/CYP1A1 pathway [100]. Therefore, these data reveal that hepatic activation of the PPAR-α pathway may represent a critical link among AhR signaling, circadian rhythms, and glucose metabolism, giving further insight into underlying AhR-mediated insulin resistance.

#### Pathological role of AhR/CYP1A1 in adipose tissue

T2DM is characterized not only by impaired secretion of insulin but also by insulin resistance among organs such as the liver, adipose tissue, and muscle majorly contribute to the metabolic condition [69]. Adipose tissues are targets for environmental toxicants in that it has been reported that AhR activators such as POPs and PCBs bioaccumulate within human and animal adipose tissues, leading to inflammation and impaired glucose homeostasis [103, 104]. Whereas administration of an AhR antagonist resveratrol prevents PCB-mediated impairment of glucose hemostasis and insulin secretion in mice adipose tissue [105]. Lee et al. reported that subjects with high blood POP levels were prone to insulin resistance and T2DM, whereas subjects with low blood POPs had no such risk, despite the onset of obesity in these subjects. This highlights the complex interaction between AhR and obesity in the pathogenesis of T2DM [104].

Numerous studies have shown that AhR is associated with the modulation of gluconeogenesis genes [106, 107]. Furthermore, an in vivo study demonstrated that intraperitoneal administration of different doses of TCDD to guinea pigs for up to 28 days caused a persistent reduction in the uptake of glucose by the pancreatic and adipose tissue, possibly owing to a decrease in the number of glucose transpor proteins [108]. Thus, it has been postulated that TCDD may directly or indirectly inhibit the transcriptional expression of glucose transporter (GLUT) genes through an AhR-dependent mechanism. Furthermore, the TCDD-induced reduction in the glucose transport system, which is known to play a vital role in supplying energy to cells and cellular metabolism, has been shown to exacerbate adipose tissue loss and inhibit lipogenesis and gluconeogenesis [108] via dysregulation of lipoprotein lipase activity in adipose tissue, insulin secretion in the pancreas, and glucose metabolism and glycogen fatty acid synthesis in the liver [109]. Taken together, these results demonstrate that TCDD exhibits inhibitory action on GLUT genes by its interaction with AhR.

**Fig. 4:**
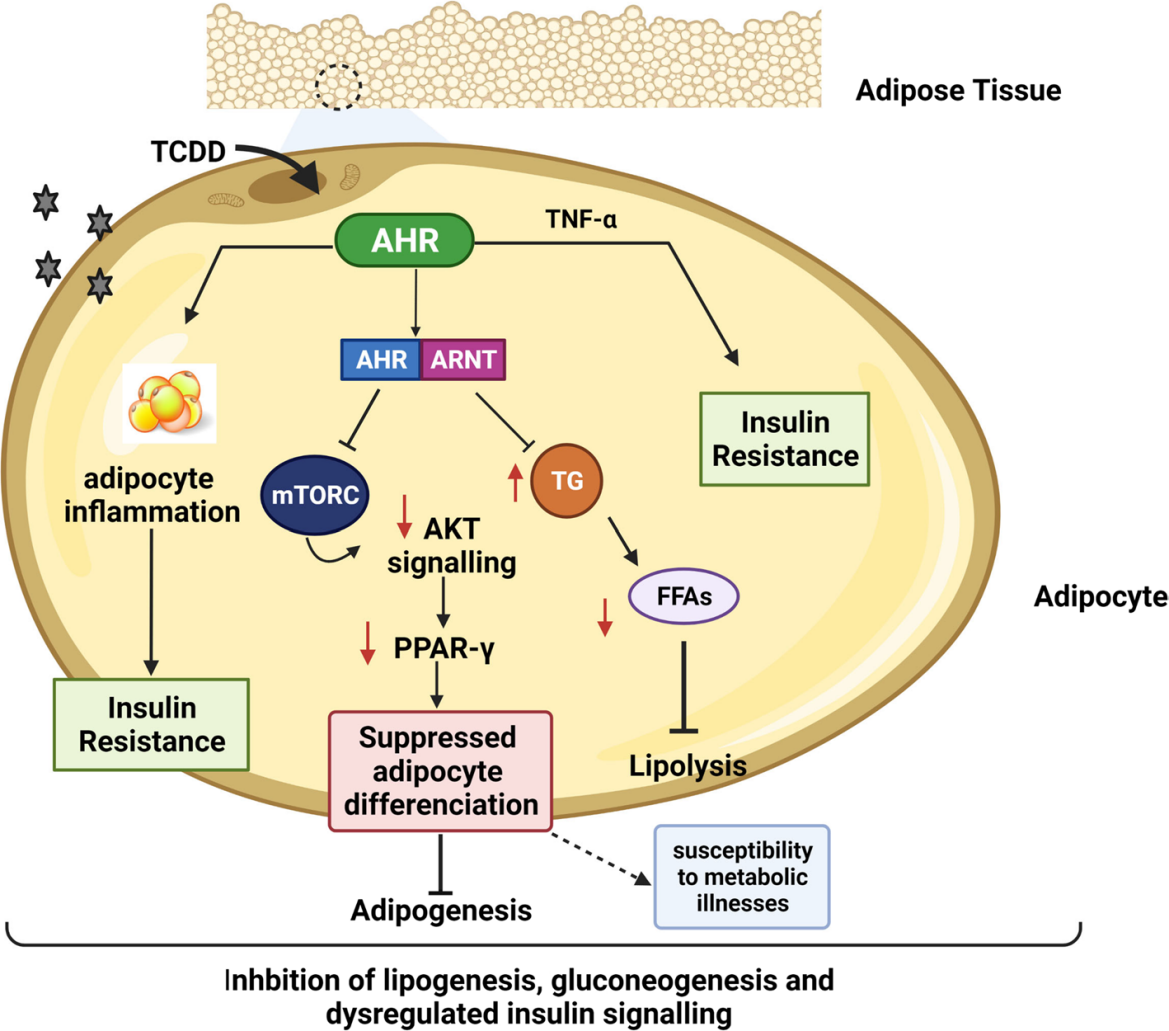
AhR regulates adipocyte differentiation by regulating the PPAR signaling pathway, which plays a vital role in modulating fatty acid oxidation and glucose metabolism. Dioxins, such as TCDD, bind to AhR, inducing receptor activation that evokes many biological and toxicological effects. These environmental toxins bioaccumulate within human and animal adipose tissues, leading to inflammation and subsequently impairing insulin resistance. Furthermore, the formation of AhR–ARNT complexes interferes with the signaling of several pathways. TCDD-induced activation of AhR further causes dysregulation of lipoprotein lipase activity in adipose tissue, regulating adipocyte differentiation and interfering in the PPAR-α signaling pathway critical in fatty acid oxidation and glucose metabolism. Additionally, a TCDD-induced increase in the expression of TNF-α has been shown to exacerbate the dysfunction of insulin signaling and insulin resistance. Created by bioRender.com

Initial studies by Poland et al. were the first to provide evidence of a link between AhR activation by TCDD and energy balance [110]. Following this, several studies demonstrated the inhibitory action of TCDD on the synthesis of fatty acids in liver and adipose tissues [111, 112], gluconeogenic enzymes such as G6Pase and PEPCK [113], and adipogenesis [114]. In this context, it has been reported that mice expressing a high constitutively active form of AhR spontaneously developed hepatic steatosis, characterized by high amounts of fatty acid import, suppressed fatty acid oxidation, and increased oxidation and mobilization of peripheral fat storage [115]. Consistent with this, Wang et al. have shown that *AhR* deletion not only stimulated insulin sensitivity, increased energy and consumption, and diminished adiposity [116], but also diminished the PPAR-α signaling pathway, which is known to play a vital role in modulating fatty acid oxidation and glucose metabolism [100]. Similarly, it has been reported that obese mice subjected to a diet containing the AhR antagonist α-NF, had a significant loss of body mass, reduced PPAR-α activity, and reversed the fatty liver disease and expression of obesity markers, such as stearoyl-CoA desaturase 1 and phosphoprotein 1 [117]. These studies strongly indicate a link between AhR and energy expenditure in experimentally induced obesity, and open doors to the potential of targeting AhR as an effective treatment strategy for obesity and related comorbidities, including DM. Figure 4 summarizes the molecular pathways that are regulated by AhR activation in the adipose tissue and the impact on glucose homeostasis and DM development. In addition, disruption of the CYP1B1 gene has demonstrated extensive protection against obesity and steatotic hepatitis [118]. In agreement with this, a study on transgenic CYP1B1 deficient mice that were fed with a high-fat diet (HFD) showed improved glucose intolerance with potentially lowered obesity compared with wild-type animals [119]. These effects of CYP1B1 on obesity and glucose intolerance could be attributed to the role of CYP1B1 in metabolizing steroid hormones and lipids that modulate metabolism, and the accumulation and distribution of adipose tissues [120, 121], suggesting that inhibition of CYP1B1 could be a biomarker in the treatment of obesity and T2DM.

## Molecular mechanisms governing the effect of AhR/CYP1A1 pathway on glucose hemostasis and DM development

The most important question is how the AhR/CYP1A1 pathway modulates glucose hemostasis and DM development. Unfortunately, there is still no clear answer to this question. However, the existing crosstalk between AhR/CYP1A1 and several transcriptional factors and molecular pathways means the role and physiological functioning of various genes have been deciphered. In general, four main mechanisms are proposed: (1) gluconeogenesis, (2) hypoxia-inducible factor (HIF), (3) oxidative stress, and (4) inflammation.

### Gluconeogenesis

Glucose is a well-known primary fuel that fulfills the energy needs of mammalian tissues. Glucose is generated through a well-orchestrated enzymatic pathway called gluconeogenesis [122]. The liver is an important site for glucose storage in the form of glycogen. In addition, it also plays a role in maintaining gluconeogenesis, that is, de novo glucose synthesis during prolonged starvation periods. However, an increase in the rate of gluconeogenesis has been greatly attributed to hyperglycemia in T2DM. The pathway is regulated by hormone secretion, gene transcription, and post-translational modification [123]. Hormones such as insulin, glucagon, and glucocorticoid modulate the gluconeogenic pathway and glucose production [123].

The involvement of the AhR pathway in gluconeogenesis was investigated in AhR knockout mice. This study showed that the expression of gluconeogenic genes such as PEPCK and G6Pase was observed in mice plasma, liver, and skeletal muscle. Furthermore, AhR knockout mice showed lower glucose levels in plasma and liver compared with AhR wild-type mice. However, this was contrary to elevated lactate levels, the main product of glycolysis, in the plasma and skeletal muscle of AhR knockout mice [124]. Since lactate, in association with alanine, functions as a critical substrate in the gluconeogenesis pathway during fasting, alanine levels were further observed and lower in AhR knockout mice muscle and liver. Therefore, increased levels of lactate combined with alanine in the plasma of AhR knockout mice could indicate glucose being catabolized by peripheral tissues and hence promote its utilization as a gluconeogenic precursor in the liver under fasting conditions for energy homeostasis [124]. Additionally, glycerol levels, a substrate for gluconeogenesis, were elevated in the plasma and liver of AhR knockout mice. These data prompted studies to reveal that the genetic expression of G6Pase was higher in AhR null mice to maintain blood glucose levels for energy metabolism under fasting conditions [124]. These results point towards the possible implications of disrupted glucose and fatty acid metabolism, such as the onset of T2DM and obesity-related comorbidities [124, 125].

### Hypoxia-inducible factor (HIF)

It was previously reported that altered insulin secretion could occur due to specific ARNT variants [83]. ARNT, known as a hypoxia-inducible factor (HIF)-1 β, is a transcription factor that plays a critical role in adaptive responses to environmental stresses, including dioxin exposure and hypoxia [126]. Therefore, an extensive study was performed to identify the functioning of metabolic pathways under low *ARNT/HIF-1β* factor levels in β-cells. In this study, siRNA-mediated knockdown of *ARNT/HIF-1β* inhibited glucose-stimulated insulin secretion. Findings from the study support the idea that ARNT/HIF-1β plays a prominent role in the regulation of biphasic insulin secretion [127].

It is also known as a joint binding partner for AhR and HIF-α subunits, and hence plays a significant role in AhR and HIF crosstalk [128, 129]. HIF signaling contributes to tumor progression by promoting invasion/metastasis, metabolic alteration, and induction of angiogenesis [126]. ARNT is expressed in pancreatic islets and plays a role in glycolysis and normal angiogenesis [83, 130]. ARNT has been found to regulate the normal function of HIF-1α [131, 132], which is important for β-cell survival and islet transplant outcomes. ARNT expression in islets of humans with T2DM was found to be reduced [38, 132, 133]. Moreover, earlier studies reveal impaired islet transplant outcomes in β-cells lacking HIF-α [134]. Hence, data from these studies implicate the importance of ARNT in β-cells and graft function, the absence of which could impair the ability of these cells to respond to physiological insults, subsequently leading to the onset of DM and unsuccessful islet transplant outcome [134, 135].

Animal knockout models have shown the crucial role of ARNT in impaired β-cell function and the pathogenesis of T2DM. Furthermore, β-cell-specific deletion of ARNT in mice has been found to significantly contribute to abnormalities in glucose metabolism and insulin secretion, alongside multiple alterations in isolated islets’ RNA expression that are highly similar to those of human pancreatic islets [38]. These observations point toward ARNT playing a role as an upstream regulator of several gene expression changes, causing the onset of T2DM in humans. In addition, since pyruvate cycling has been proposed as a novel pathway in insulin release [136], 137, its key NADP+ dependent malic enzyme (MEc) and pyruvate carboxylase (PC) were downregulated, suggesting the role of ARNT/HIF-1β in pyruvate cycling [127]. Collectively, there was a marked reduction in the glycolytic flux in *ARNT/HIF-1*β silenced β-cells, highlighting the absolute importance of ARNT/HIF-1β in maintaining the glucose-responsive state of pancreatic β-cells and insulin release.

### Oxidative stress

Several studies have recognized oxidative stress as a critical marker for developing diabetic complications [138–141]. Oxidative stress occurs from the unbalanced production of free oxygen radicals and ROS that can cause DNA, RNA, and protein damage, in addition to cellular membrane destruction [142]. The onset of diabetes has shown increased levels of oxidative stress in the β-cells, a phenomenon caused by elevated production of oxidants and an impaired antioxidant defense system. This imbalance could be attributed to several mechanisms, including the increased intracellular formation of ROS, lower ATP/ADP ratio, reduction of the mitochondrial membrane potential, and downregulation of expression of genes associated with energy metabolism [143–145]. Such effects lead to cellular and tissue damage, specifically the development of diabetic complications such as diabetic nephropathy and cardiomyopathy [146]. Owing to the high endogenous production of ROS and decreased antioxidant capacity, islets of the pancreas are highly susceptible to oxidative stress, making it a plausible reason for beta cell failure [147].

To further understand critical mechanisms governing this metabolic condition, numerous studies have investigated the association of oxidative stress and AhR activity, including CYP1 enzymes [148]. A previous study investigated the pathological role of AhR in diabetic nephropathy in in vitro and in vivo models, and revealed that AhR deficiency attenuates oxidative stress in both models. In addition, hyperglycemia and glucose intolerance induced by STZ, a well-known experimental inducer of hyperglycemia, were attenuated in AhR knockout, which was associated with lower expression of cyclooxygenase (COX-2) in the kidneys under diabetic conditions, implicating the critical role of AhR in the pathogenesis of diabetic nephropathy [149]. Furthermore, the AhR antagonist, alizarin, prevented alloxan-induced DM and oxidative stress in mice [150]. The CYP1 enzymes are involved in the oxidative metabolism of xenobiotic agents via utilizing molecular oxygen for the combined oxidation of NADPH; therefore, they are a potential source of ROS generation [151]. This was supported by a study demonstrated that mice treated with TCDD had elevated levels of ROS in the liver mitochondria, a prime site for energy disposition and dysfunction [152], which could lead to hepatic insulin resistance [152, 153].

### Inflammation

A notable correlation between obesity and T2DM and chronic low-grade systemic inflammation, which could be responsible for the onset of insulin resistance and progression of diabetes complications, has also been demonstrated in animal models and patients with T2DM and obesity [154–158]. Previous studies have revealed a significant elevation in the Th17 and Th1 subset of cells, along with a decrease in the Treg subgroup in patients with T2DM and obesity [154, 155]. Additionally, serum levels of proinflammatory cytokines, including IL-7, IL-22, and IL-6, were significantly high in T2DM patients, suggesting an aggravated inflammatory status. Interestingly, in diabetic patients, the number and function of immune cells are distorted, including innate and adaptive immunity [155]. Accumulating evidence also established that immune cells, especially T lymphocyte alterations, preceded the loss of insulin sensitivity in adipose tissue and contributed to the general proinflammatory drift observed in obesity and T2DM [155, 158].

Numerous studies over the last decade highlight the emerging role of AhR in disease modulation, specifically in regulating immune responses and inflammation [159]. It has been reported that AhR expression in the adipose tissue significantly mediates adipose tissue inflammation and impairment of glucose hemostasis in obese mice [160]. A recent study has demonstrated an increase in AhR mRNA expression levels in peripheral blood mononuclear cells (PBMCs) from patients with T2DM and metabolically healthy obese patients, which was correlated with elevated levels of the proinflammatory IL-22 and LI-17 levels. In this study, AhR transcripts were greatly correlated with insulin resistance and basal β-cell function, indicating a role of AhR-mediated inflammation in the development of obesity and T2DM [161]. Similarly, macrophages, another set of immune cells, demonstrate a prominent role in T1DM initiation and progression through antigen presentation or producing inflammatory cytokines that destroy β-cells [162]. Hence, in conjunction with this, AhR, upon activation, reduces IL-6 expression in macrophages, diminishing uncontrolled inflammatory responses [163]. In addition, a large-scale gene expression analysis was conducted in vitro in human adipose-derived stem cells and in vivo mice to explore the role of AhR ligands on inflammatory gene expression in adipocytes. The study revealed that AhR ligands significantly induced inflammatory markers IL-8 and monocyte chemoattractant protein-1 and CYP1B1 in adipose tissues, which was reduced by pretreatment with α-NF, an AhR antagonist [164].

## Paradoxical role of AhR/CYP1A1 pathway in DM

AhR is known for its multifaceted role in association with ligand type, tissue specificity, and disease models. Contrary to what was discussed earlier in this review, a few studies have reported the anti-diabetogenic role of the AhR/CYP1A pathway. This section highlights these studies and their main findings.

TCDD, the most potent agonist of AhR, is known for its immunosuppressive effects. However, more recently, the AhR receptor is gaining momentum for its protective role in various physiological mechanisms. An experimental study on a mice model was carried out to determine the effect of chronic treatment with TCDD on disease development and CD4^+^ T cells [165]. NOD mice treated with a biweekly TCDD dosing regimen over a 24-week experimental period exhibited complete suppression of diabetes development in 100% of the animals through 31 weeks. However, at 23 weeks of age, halting TCDD treatment initiated the onset of diabetes in 50% of mice. This means that as the body burden of TCDD declined to levels that could not activate AhR, *Cyp1a1* gene expression within the liver diminished, leading to diabetes initiation within these mice [165].

Furthermore, pancreatic lymph nodes of TCDD-treated mice demonstrated an increased frequency of CD4^+^ CD25^+^ Foxp3^+^ T-cells. This could possibly be because AhR in CD4^+^ T cells may have increased the frequency of Treg cells, suppressing the development of diabetes [165]. Therefore, it is imperative to understand further the mechanisms that govern AhR-mediated effects on Treg development and functioning. This may open new avenues for treating T1DM and other immune-related diseases. However, it remains unclear how different agonistic AhR ligands alter the receptor’s behavior. A multitude of factors could be attributed to this; for instance, different ligand-binding with AhR alters the receptor's conformation, allowing the recruitment of unique co-activators or co-repressors and therefore altering gene expression. This could be an important area of research for further study.

The hypoglycemia as one of the consequences of TCDD toxicity is supported by the experimental study on STZ-induced diabetic rats treated with TCDD [166, 167]. In this study, TCDD treatment successfully reversed hyperglycemia in the STZ-induced T2DM rat model [168]. However, the precise mechanism leading to these observations has not been fully explored. A longitudinal birth cohort study with follow-up into the adolescent stage, found key links between perinatal dioxin exposure and disrupted glucose metabolism and insulin secretion in later stages of life [169, 170]. The outcome of these studies highlighted that prenatal TCDD exposure was linked to lower insulin resistance and β-cell challenge among female offspring but not in males [170]. This could suggest a gender-specific influence of dioxin exposure in the perinatal period on pancreatic development [170]. In addition, a natural AhR agonist, Indigo, protects against HFD-induced insulin resistance, glucose dysregulation, and fatty liver disease in an HFD-induced animal model [171].

## Conclusions

The prevalence of DM has greatly increased in the past decade and is a global public health concern. AhR has long been recognized as an evolutionarily conserved ligand-activated transcription factor, best known for its role in xenobiotic metabolism. AhR signaling is driven by environmental signals and endogenous metabolites that are of critical importance for the maintenance of several functions in the body, such as hormone levels, circadian clock, gastrointestinal homeostasis, and cell proliferation. In recent years, AhR has been shown to be an important modulator of disease, with numerous studies establishing that exposure to xenobiotic AhR ligands could contribute to the growing incidence of metabolic conditions such as DM. Additionally, TCDD, a potent environmental contaminant, has a high binding affinity to AhR and is thus being used to extensively study the receptor-associated activation of various physiological functions in the body. A growing body of evidence highlights that toxicant-activated AhR signaling disrupts fat metabolism, glucose homeostasis, and insulin secretion, thus causing metabolic dysfunction. Furthermore, over-activation of the receptor has been found to promote hepatic steatosis, the onset of insulin resistance, causing glucose intolerance and eventually leading to diabetes. Collectively, these studies demonstrate that an intrinsic increase in the expression of AhR and its activity through endogenous and exogenous ligand factors has the potential to exert multifaceted influences on the pathophysiology of metabolic disorders, including DM.

In this review, we highlighted recent developments that point toward the role AhR may have on metabolism and, thus in the development of diabetes. Furthermore, understanding the integrated network of AhR and its XMEs, such as *CYP1A1*, in signaling pathways within organs such as the liver, pancreas (β-cell), and adipose tissues may shed light on the possible physiological activators of AhR in DM. Paradoxically, other studies have demonstrated the antidiabetogenic effect of AhR/CYP1A pathway, depending on its association with the ligand type, tissue specificity, and disease models. Table 1 summarizes all the studies exporting the role of AhR/CYP1 pathway in glucose homeostasis, insulin resistance, and diabetes development in human and different animal models.In conclusion, further evaluation of the mechanisms governing AhR effect on diabetes will give further insights into understanding the disease and pave the way for targeted pharmaceuticals and therapeutics.

**Table 1:** Effect of AhR/CYP1A1 pathways on various species under diabetic conditions

DM		Species	Treatment	Effect of AhR/CYP1A1 pathway in glucose hemostasis, insulin secretion, and DM	References
T1DM	Mice	Mice	NOD-mice	Pancreatic endocrine cells appear in crosstalk with gut microbiota via AMPs AMPs halt pancreatic inflammation AhR was expressed in intestinal ILCs, establishing that AhR–ILC axis exists to maintain gut integrity, which impacts T1DM progression	[58, 60]
		C57BL/6 mice	TCDD	AhR activation promoted Treg induction and development by binding to the Foxp3 + promoter	[53]
T2DM	Human	Pancreatic endocrine cell line	TCDD	**↓** insulin secretion **↓** plasma insulin level **↑** β-cells death	[46]
		hESC cells	Low dose TCDD	Impaired pancreatic lineage differentiation and AMPK pathway leading to impaired pancreatic development and function	[70]
		Aortic endothelial cells (ECs)	High glucose levels	**↑** AhR activity leads to increased TSP-1, which is involved in the development of diabetic vascular complications AhR interacts with Egr-1 and AP-2 → endothelial dysfunction and microvascular complications	[72]
		Human Sera	Serum TCDD levels	Korean diabetic patients have higher serum AhR ligand TCDD levels > non-diabetics mitochondrial dysfunction leads to the pathogenesis of insulin resistance	[76, 80]
	Mice	Hepa-1c1c7 (cell line)	β-NF, TCDD,	**↑** insulin sensitivity, **↓** PPAR-α expression and altered G6Pase and PEPCK in AhR KO mice **↑** Cyp1a1 and PPAR-α expression	[102]
		Mice	TCDD	**↓** G6Pase & PEPCK enzymes **↓** adipogenesis **↑** ROS levels in the liver mitochondria leading to hepatic insulin resistance	[113, 114, 152,], [172]
		Obese mice	α-NF	**↓** PPAR-α activity expression of obesity markers, stearoyl-CoA desaturase one and phosphoprotein 1	[117]
		Mice	AhR KO	**↓** glucose levels in plasma and liver **↓** alanine levels (substrate in the gluconeogenesis during fasting), muscle and liver disrupted glucose and fatty acid metabolisms	[124]
		Mice	STZ	**↓** expression of COX-2 in the kidneys under diabetic conditions, thus highlighting a link between AhR and diabetic nephropathy	[149]
		Kunming diabetic mice	Alizarin	**↓** glucose levels, lipid profile, and oxidative stress	[150]
		C57BL/6	PCBs α-NF, resveratrol	**↑** impairment of glucose and insulin **↑** inflammatory mediators, TNF-α in adipose tissue **↓** glucose intolerance and insulin secretion	[103] [103, 105]
	Guinea pigs	Guinea pigs	Transient TCDD exposure	**↓** glucose uptake in the pancreas and adipose tissue, owing to the reduced number of glucose transporting proteins on the plasma membrane of these organs **↓** GLUT genes via AhR-dependent mechanism **↓** lipogenesis & gluconeogenesis, dysregulated lipoprotein lipase activity, disrupted insulin production and fatty acid synthesis	[108, 109]
	Rats	Sprague–Dawley	Treatment with STZ	**↑** DM associated with increased CYP1A1 activity levels	[173]

## Data Availability

No data was used for the research described in the article.

## Abbreviations

3MC: 3-Methylcholanthrene
AP-2: Activator protein-2
AhR: Aryl hydrocarbon receptor
ARNT: AhR nuclear translocator
bHLH: Basic helix-loop-helix
CYP: Cytochrome P450 proteins
CYP1A1: Cytochrome P450 proteins 1A1
CYP1B1: Cytochrome P450 proteins 1B1
DMBA: 7,12-Dimethybenz[a]anthracene
EGR-1: Early growth response
EROD: Ethoxy resorufin *O*-deethylase
G6Pase: Glucose-6-phosphatase
Hif-α: Hypoxia-inducible factor
HSP90: Heat shock protein 90
IFN-γ: Interferon-γ
DM: Diabetes mellitus
MODY-2: Mature-onset diabetes of the young-2
NPBMCs: Peripheral blood mononuclear cells
PAHs: Polycyclic aromatic hydrocarbons
PAS: Period/ARNT/single
PCBs: Polychlorinated biphenyls
PM: Particulate matter
POPs: Persistent organic pollutants
PPAR-α: Peroxisome proliferator-activated receptor
ROS: Reactive oxygen species
STZ: Streptozotocin
T1DM: Type 1 diabetes mellitus
T2DM: Type 2 diabetes mellitus
TCDD: 2,3,7,8-Tetrachlorodibenzo-*p*-dioxin
TNF-α: Tumor necrosis factor
TSP-1: Thrombospondin-1
XRE: Xenobiotic responsive elements

## References

[1] Guariguata L, Whiting DR, Hambleton I, Beagley J, Linnenkamp U, Shaw JE (2014). Global estimates of diabetes prevalence for 2013 and projections for 2035. Diabetes Res Clin Pract.

[2] Chawla R, Madhu SV, Makkar BM, Ghosh S, Saboo B, Kalra S (2020). Group R-EC: RSSDI-ESI clinical practice recommendations for the management of type 2 diabetes mellitus 2020. Indian J Endocrinol Metab.

[3] Prasad H, Ryan DA, Celzo MF, Stapleton D (2012). Metabolic syndrome: definition and therapeutic implications. Postgrad Med.

[4] Fuller R, Landrigan PJ, Balakrishnan K, Bathan G, Bose-O'Reilly S, Brauer M, Caravanos J, Chiles T, Cohen A, Corra L (2022). Pollution and health: a progress update. Lancet Planet Health.

[5] Kerzee JK, Ramos KS (2001). Constitutive and inducible expression of Cyp1a1 and Cyp1b1 in vascular smooth muscle cells: role of the Ahr bHLH/PAS transcription factor. Circ Res.

[6] Whitelaw ML, Gottlicher M, Gustafsson JA, Poellinger L (1993). Definition of a novel ligand binding domain of a nuclear bHLH receptor: co-localization of ligand and hsp90 binding activities within the regulable inactivation domain of the dioxin receptor. Embo J.

[7] Kawajiri K, Fujii-Kuriyama Y (2017). The aryl hydrocarbon receptor: a multifunctional chemical sensor for host defense and homeostatic maintenance. Exp Anim.

[8] Dhulkifle H, Agouni A, Zeidan A, Al-Kuwari MS, Parray A, Tolefat M, Korashy HM (2021). Influence of the aryl hydrocarbon receptor activating environmental pollutants on autism spectrum disorder. Int J Mol Sci.

[9] Akhtar S, Hourani S, Therachiyil L, Al-Dhfyan A, Agouni A, Zeidan A, Uddin S, Korashy HM (2020). Epigenetic regulation of cancer stem cells by the aryl hydrocarbon receptor pathway. Semin Cancer Biol.

[10] Fujii-Kuriyama Y, Ema M, Mimura J, Matsushita N, Sogawa K (1995). Polymorphic forms of the Ah receptor and induction of the CYP1A1 gene. Pharmacogenetics.

[11] Shimada T, Fujii-Kuriyama Y (2004). Metabolic activation of polycyclic aromatic hydrocarbons to carcinogens by cytochromes P450 1A1 and 1B1. Cancer Sci.

[12] Beyersmann D (2002). Effects of carcinogenic metals on gene expression. Toxicol Lett.

[13] ATSDR: Agency for Toxic Substances and Disease Registry, The ATSDR 2019 Substance Priority List. Public Health Service; https://www.atsdrcdcgov/spl/ 2019.

[14] Dimakakou E, Johnston HJ, Streftaris G, Cherrie JW (2018). Exposure to environmental and occupational particulate air pollution as a potential contributor to neurodegeneration and diabetes: a systematic review of epidemiological research. Int J Environ Res Public Health.

[15] Hernandez AM, Gimeno Ruiz de Porras D, Marko D, Whitworth KW (2018). The association between PM2.5 and ozone and the prevalence of diabetes mellitus in the United States, 2002 to 2008. J Occup Environ Med.

[16] Hwang MJ, Kim JH, Koo YS, Yun HY, Cheong HK (2020). Impacts of ambient air pollution on glucose metabolism in Korean adults: a Korea National Health and Nutrition Examination Survey study. Environ Health.

[17] Chen Z, Salam MT, Toledo-Corral C, Watanabe RM, Xiang AH, Buchanan TA, Habre R, Bastain TM, Lurmann F, Wilson JP (2016). Ambient air pollutants have adverse effects on insulin and glucose homeostasis in Mexican Americans. Diabetes Care.

[18] Duncan BB, Castilhos CD, Bracco PA, Schmidt MI, Kang S, Im S, Lee HK, Vigo A, Pak YK (2020). Aryl-hydrocarbon receptor binding and the incidence of type 2 diabetes: the Brazilian Longitudinal Study of Adult Health (ELSA-Brasil). Environ Health.

[19] Thayer KA, Heindel JJ, Bucher JR, Gallo MA (2012). Role of environmental chemicals in diabetes and obesity: a National Toxicology Program workshop review. Environ Health Perspect.

[20] Lee DH, Lind L, Jacobs DR, Salihovic S, van Bavel B, Lind PM (2012). Associations of persistent organic pollutants with abdominal obesity in the elderly: the Prospective Investigation of the Vasculature in Uppsala Seniors (PIVUS) study. Environ Int.

[21] Lind PM, Risérus U, Salihovic S, Bavel B, Lind L (2013). An environmental wide association study (EWAS) approach to the metabolic syndrome. Environ Int.

[22] Lee DH, Steffes M, Jacobs DR (2007). Positive associations of serum concentration of polychlorinated biphenyls or organochlorine pesticides with self-reported arthritis, especially rheumatoid type, in women. Environ Health Perspect.

[23] Fierens S, Mairesse H, Heilier JF, De Burbure C, Focant JF, Eppe G, De Pauw E, Bernard A (2003). Dioxin/polychlorinated biphenyl body burden, diabetes and endometriosis: findings in a population-based study in Belgium. Biomarkers.

[24] Longnecker MP, Klebanoff MA, Brock JW, Zhou H (2001). Polychlorinated biphenyl serum levels in pregnant subjects with diabetes. Diabetes Care.

[25] Radikova Z, Koska J, Ksinantova-Hornanska L, Imrich R, Kocan A, Petrik J, Huckova M, Wsólová L, Langer P, Trnovec T (2004). Increased frequency of diabetes and other forms of dysglycemia in the population of specific areas of eastern Slovakia chronically exposed to contamination with polychlorinated biphenyls (PCB). Organohalogen Compd.

[26] Vasiliu O, Cameron L, Gardiner J, Deguire P, Karmaus W (2006). Polybrominated biphenyls, polychlorinated biphenyls, body weight, and incidence of adult-onset diabetes mellitus. Epidemiology.

[27] Codru N, Schymura MJ, Negoita S, Rej R, Carpenter DO (2007). Diabetes in relation to serum levels of polychlorinated biphenyls and chlorinated pesticides in adult Native Americans. Environ Health Perspect.

[28] Walisser JA, Bunger MK, Glover E, Bradfield CA (2004). Gestational exposure of Ahr and Arnt hypomorphs to dioxin rescues vascular development. Proc Natl Acad Sci USA.

[29] Vasquez A, Atallah-Yunes N, Smith FC, You X, Chase SE, Silverstone AE, Vikstrom KL (2003). A role for the aryl hydrocarbon receptor in cardiac physiology and function as demonstrated by AhR knockout mice. Cardiovasc Toxicol.

[30] Barouki R, Coumoul X, Fernandez-Salguero PM (2007). The aryl hydrocarbon receptor, more than a xenobiotic-interacting protein. FEBS Lett.

[31] Puga A, Ma C, Marlowe JL (2009). The aryl hydrocarbon receptor crosstalks with multiple signal transduction pathways. Biochem Pharmacol.

[32] McMillan BJ, Bradfield CA (2007). The Aryl hydrocarbon receptor is activated by modified low-density lipoprotein. Proc Natl Acad Sci.

[33] Henry DJ, Greene MA, White FJ (1989). Electrophysiological effects of cocaine in the mesoaccumbens dopamine system: repeated administration. J Pharmacol Exp Ther.

[34] Swanson HI, Bradfield CA (1993). The AH-receptor: genetics, structure and function. Pharmacogenetics.

[35] Hahn ME, Karchner SI, Shapiro MA, Perera SA (1997). Molecular evolution of two vertebrate aryl hydrocarbon (dioxin) receptors (AHR1 and AHR2) and the PAS family. Proc Natl Acad Sci USA.

[36] Carver LA, Hogenesch JB, Bradfield CA (1994). Tissue specific expression of the rat Ah-receptor and ARNT mRNAs. Nucleic Acids Res.

[37] Koliopanos A, Kleeff J, Xiao Y, Safe S, Zimmermann A, Büchler MW, Friess H (2002). Increased arylhydrocarbon receptor expression offers a potential therapeutic target for pancreatic cancer. Oncogene.

[38] Gunton JE, Kulkarni RN, Yim S, Okada T, Hawthorne WJ, Tseng YH, Roberson RS, Ricordi C, O'Connell PJ, Gonzalez FJ (2005). Loss of ARNT/HIF1beta mediates altered gene expression and pancreatic-islet dysfunction in human type 2 diabetes. Cell.

[39] Clarke J, Flatt PR, Barnett CR (1997). Cytochrome P450 1A-like proteins expressed in the islets of Langerhans and altered pancreatic beta-cell secretory responsiveness. Br J Pharmacol.

[40] Falck JR, Manna S, Moltz J, Chacos N, Capdevila J (1983). Epoxyeicosatrienoic acids stimulate glucagon and insulin release from isolated rat pancreatic islets. Biochem Biophys Res Commun.

[41] Foster JR, Idle JR, Hardwick JP, Bars R, Scott P, Braganza JM (1993). Induction of drug-metabolizing enzymes in human pancreatic cancer and chronic pancreatitis. J Pathol.

[42] Lee YM, Ha CM, Kim SA, Thoudam T, Yoon YR, Kim DJ, Kim HC, Moon HB, Park S, Lee IK (2017). Low-dose persistent organic pollutants impair insulin secretory function of pancreatic β-Cells: human and in vitro evidence. Diabetes.

[43] Mailloux R, Fu A, Florian M, Petrov I, Chen Q, Coughlan MC, Laziyan M, Yan J, Caldwell D, Patry D (2015). A Northern contaminant mixture impairs pancreas function in obese and lean JCR rats and inhibits insulin secretion in MIN6 cells. Toxicology.

[44] Novelli M, Piaggi S, De Tata V (2005). 2,3,7,8-Tetrachlorodibenzo-p-dioxin-induced impairment of glucose-stimulated insulin secretion in isolated rat pancreatic islets. Toxicol Lett.

[45] Kurita H, Yoshioka W, Nishimura N, Kubota N, Kadowaki T, Tohyama C (2009). Aryl hydrocarbon receptor-mediated effects of 2,3,7,8-tetrachlorodibenzo-*p*-dioxin on glucose-stimulated insulin secretion in mice. J Appl Toxicol.

[46] Ibrahim M, MacFarlane EM, Matteo G, Hoyeck MP, Rick KRC, Farokhi S, Copley CM, O’Dwyer S, Bruin JE (2020). Functional cytochrome P450 1A enzymes are induced in mouse and human islets following pollutant exposure. Diabetologia.

[47] Thackaberry EA, Bedrick EJ, Goens MB, Danielson L, Lund AK, Gabaldon D, Smith SM, Walker MK (2003). Insulin regulation in AhR-null Mice: embryonic cardiac enlargement, neonatal macrosomia, and altered insulin regulation and response in pregnant and aging AhR-null females. Toxicol Sci.

[48] Biljes D, Hammerschmidt-Kamper C, Kadow S, Diel P, Weigt C, Burkart V, Esser C (2015). Impaired glucose and lipid metabolism in ageing aryl hydrocarbon receptor deficient mice. EXCLI J.

[49] Buchanan TA, Metzger BE, Freinkel N, Bergman RN (1990). Insulin sensitivity and B-cell responsiveness to glucose during late pregnancy in lean and moderately obese women with normal glucose tolerance or mild gestational diabetes. Am J Obstet Gynecol.

[50] Spellacy WN, Miller S, Winegar A, Peterson PQ (1985). Macrosomia–maternal characteristics and infant complications. Obstet Gynecol.

[51] Lim CC, Thurston GD (2019). Air pollution, oxidative stress, and diabetes: a life course epidemiologic perspective. Curr Diab Rep.

[52] de Lima KA, Donate PB, Talbot J, Davoli-Ferreira M, Peres RS, Cunha TM, Alves-Filho JC, Cunha FQ (2018). TGFβ1 signaling sustains aryl hydrocarbon receptor (AHR) expression and restrains the pathogenic potential of TH17 cells by an AHR-independent mechanism. Cell Death Dis.

[53] Mezrich JD, Fechner JH, Zhang X, Johnson BP, Burlingham WJ, Bradfield CA (2010). An interaction between kynurenine and the aryl hydrocarbon receptor can generate regulatory T cells. J Immunol.

[54] Rodríguez-Sosa M, Elizondo G, López-Durán RM, Rivera I, Gonzalez FJ, Vega L (2005). Over-production of IFN-gamma and IL-12 in AhR-null mice. FEBS Lett.

[55] Maltepe E, Schmidt JV, Baunoch D, Bradfield CA, Simon MC (1997). Abnormal angiogenesis and responses to glucose and oxygen deprivation in mice lacking the protein ARNT. Nature.

[56] Yue T, Sun F, Yang C, Wang F, Luo J, Yang P, Xiong F, Zhang S, Yu Q, Wang C-Y (2020). The AHR signaling attenuates autoimmune responses during the development of type 1 diabetes. Front Immunol.

[57] Saxena V, Ondr JK, Magnusen AF, Munn DH, Katz JD (2007). The countervailing actions of myeloid and plasmacytoid dendritic cells control autoimmune diabetes in the nonobese diabetic mouse. J Immunol.

[58] Miani M, Le Naour J, Waeckel-Enée E, Verma SC, Straube M, Emond P, Ryffel B, van Endert P, Sokol H, Diana J (2018). Gut microbiota-stimulated innate lymphoid cells support β-Defensin 14 expression in pancreatic endocrine cells. Prev Autoimmune Diabetes Cell Metab.

[59] Chen Y-G, Mathews CE, Driver JP (2018). The role of NOD mice in type 1 diabetes research: lessons from the past and recommendations for the future. Front Endocrinol.

[60] Lee JS, Cella M, McDonald KG, Garlanda C, Kennedy GD, Nukaya M, Mantovani A, Kopan R, Bradfield CA, Newberry RD (2011). AHR drives the development of gut ILC22 cells and postnatal lymphoid tissues via pathways dependent on and independent of Notch. Nat Immunol.

[61] Zenewicz LA, Flavell RA (2008). IL-22 and inflammation: Leukin' through a glass onion. Eur J Immunol.

[62] Sakaguchi S (2004). Naturally arising CD4+ regulatory t cells for immunologic self-tolerance and negative control of immune responses. Annu Rev Immunol.

[63] Fontenot JD, Gavin MA, Rudensky AY (2003). Foxp3 programs the development and function of CD4+CD25+ regulatory T cells. Nat Immunol.

[64] Hori S, Nomura T, Sakaguchi S (2003). Control of regulatory T cell development by the transcription factor Foxp3. Science.

[65] Chen W, Jin W, Hardegen N, Lei KJ, Li L, Marinos N, McGrady G, Wahl SM (2003). Conversion of peripheral CD4+CD25- naive T cells to CD4+CD25+ regulatory T cells by TGF-beta induction of transcription factor Foxp3. J Exp Med.

[66] Quintana FJ, Basso AS, Iglesias AH, Korn T, Farez MF, Bettelli E, Caccamo M, Oukka M, Weiner HL (2008). Control of T(reg) and T(H)17 cell differentiation by the aryl hydrocarbon receptor. Nature.

[67] Goettel JA, Gandhi R, Kenison JE, Yeste A, Murugaiyan G, Sambanthamoorthy S, Griffith AE, Patel B, Shouval DS, Weiner HL (2016). AHR activation is protective against colitis driven by T cells in humanized mice. Cell Rep.

[68] Fajans SS, Bell GI, Polonsky KS (2001). Molecular mechanisms and clinical pathophysiology of maturity-onset diabetes of the young. N Engl J Med.

[69] Porte D (2001). Clinical importance of insulin secretion and its interaction with insulin resistance in the treatment of type 2 diabetes mellitus and its complications. Diabetes Metab Res Rev.

[70] Kubi JA, Chen ACH, Fong SW, Lai KP, Wong CKC, Yeung WSB, Lee KF, Lee YL (2019). Effects of 2,3,7,8-tetrachlorodibenzo-p-dioxin (TCDD) on the differentiation of embryonic stem cells towards pancreatic lineage and pancreatic beta cell function. Environ Int.

[71] Kuzgun G, Basaran R, Arioglu Inan E, Can Eke B (2020). Effects of insulin treatment on hepatic CYP1A1 and CYP2E1 activities and lipid peroxidation levels in streptozotocin-induced diabetic rats. J Diabetes Metab Disord.

[72] Dabir P, Marinic TE, Krukovets I, Stenina OI (2008). Aryl hydrocarbon receptor is activated by glucose and regulates the thrombospondin-1 gene promoter in endothelial cells. Circ Res.

[73] Nathan DM, Genuth S, Lachin J, Cleary P, Crofford O, Davis M, Rand L, Siebert C (1993). The effect of intensive treatment of diabetes on the development and progression of long-term complications in insulin-dependent diabetes mellitus. N Engl J Med.

[74] Kim JT, Kim SS, Jun DW, Hwang YH, Park W-H, Pak YK, Lee HK (2013). Serum arylhydrocarbon receptor transactivating activity is elevated in type 2 diabetic patients with diabetic nephropathy. J Diabetes Investig.

[75] Kern PA, Fishman RB, Song W, Brown AD, Fonseca V (2002). The effect of 2,3,7,8-tetrachlorodibenzo-p-dioxin (TCDD) on oxidative enzymes in adipocytes and Liver. Toxicology.

[76] Park WH, Jun DW, Kim JT, Jeong JH, Park H, Chang YS, Park KS, Lee HK, Pak YK (2013). Novel cell-based assay reveals associations of circulating serum AhR-ligands with metabolic syndrome and mitochondrial dysfunction. BioFactors.

[77] Masoudi S, Nemati A, Fazli H, Beygi S, Moradzadeh M, Pourshams A, Mohamadkhani A (2018). An increased level of Aryl hydrocarbon receptor in patients with pancreatic cancer. Middle East J Dig Dis.

[78] Eckers A, Jakob S, Heiss C, Haarmann-Stemmann T, Goy C, Brinkmann V, Cortese-Krott MM, Sansone R, Esser C, Ale-Agha N (2016). The aryl hydrocarbon receptor promotes aging phenotypes across species. Sci Rep.

[79] Kirkman MS, Briscoe VJ, Clark N, Florez H, Haas LB, Halter JB, Huang ES, Korytkowski MT, Munshi MN, Odegard PS (2012). Diabetes in older adults. Diabetes Care.

[80] Johannsen DL, Ravussin E (2009). The role of mitochondria in health and disease. Curr Opin Pharmacol.

[81] Niki I, Niwa T, Yu W, Budzko D, Miki T, Senda T (2003). Ca2+ influx does not trigger glucose-induced traffic of the insulin granules and alteration of their distribution. Exp Biol Med.

[82] Kim S-Y, Lee H-G, Choi E-J, Park K-Y, Yang J-H (2007). TCDD alters PKC signaling pathways in developing neuronal cells in culture. Chemosphere.

[83] Das SK, Sharma NK, Chu WS, Wang H, Elbein SC (2008). Aryl hydrocarbon receptor nuclear translocator (ARNT) gene as a positional and functional candidate for type 2 diabetes and prediabetic intermediate traits: mutation detection, case–control studies, and gene expression analysis. BMC Med Genet.

[84] Stenina OI, Krukovets I, Wang K, Zhou Z, Forudi F, Penn MS, Topol EJ, Plow EF (2003). Increased expression of thrombospondin-1 in vessel wall of diabetic Zucker rat. Circulation.

[85] Poland A, Knutson JC (1982). 2,3,7,8-tetrachlorodibenzo-p-dioxin and related halogenated aromatic hydrocarbons: examination of the mechanism of toxicity. Annu Rev Pharmacol Toxicol.

[86] Whitlock JP (1987). The regulation of gene expression by 2,3,7,8-tetrachlorodibenzo-p-dioxin. Pharmacol Rev.

[87] Birnbaum LS (1994). Evidence for the role of the Ah receptor in response to dioxin. Prog Clin Biol Res.

[88] Barnett CR, Flatt PR, Ioannides C (1990). Induction of hepatic microsomal P450 I and IIB proteins by hyperketonaemia. Biochem Pharmacol.

[89] Barnett CR, Rudd S, Flatt PR, Ioannides C (1993). Sex differences in the diabetes-induced modulation of rat hepatic cytochrome P450 proteins. Biochem Pharmacol.

[90] Abbott BD, Probst MR (1995). Developmental expression of two members of a new class of transcription factors: II. Expression of aryl hydrocarbon receptor nuclear translocator in the C57BL/6N mouse embryo. Dev Dyn.

[91] Andreola F, Hayhurst GP, Luo G, Ferguson SS, Gonzalez FJ, Goldstein JA, De Luca LM (2004). Mouse liver CYP2C39 is a novel retinoic acid 4-hydroxylase. Its down-regulation offers a molecular basis for liver retinoid accumulation and fibrosis in aryl hydrocarbon receptor-null mice. J Biol Chem.

[92] Andreola F, Fernandez-Salguero PM, Chiantore MV, Petkovich MP, Gonzalez FJ, De Luca LM (1997). Aryl hydrocarbon receptor knockout mice (AHR-/-) exhibit liver retinoid accumulation and reduced retinoic acid metabolism. Cancer Res.

[93] Oström M, Loffler KA, Edfalk S, Selander L, Dahl U, Ricordi C, Jeon J, Correa-Medina M, Diez J, Edlund H (2008). Retinoic acid promotes the generation of pancreatic endocrine progenitor cells and their further differentiation into beta-cells. PLoS ONE.

[94] Marcheva B, Ramsey KM, Buhr ED, Kobayashi Y, Su H, Ko CH, Ivanova G, Omura C, Mo S, Vitaterna MH (2010). Disruption of the clock components CLOCK and BMAL1 leads to hypoinsulinaemia and diabetes. Nature.

[95] Oishi K, Shirai H, Ishida N (2005). CLOCK is involved in the circadian transactivation of peroxisome-proliferator-activated receptor alpha (PPARalpha) in mice. Biochem J.

[96] Turek FW, Joshu C, Kohsaka A, Lin E, Ivanova G, McDearmon E, Laposky A, Losee-Olson S, Easton A, Jensen DR (2005). Obesity and metabolic syndrome in circadian Clock mutant mice. Science.

[97] Peters JM, Shah YM, Gonzalez FJ (2012). The role of peroxisome proliferator-activated receptors in carcinogenesis and chemoprevention. Nat Rev Cancer.

[98] Canaple L, Rambaud J, Dkhissi-Benyahya O, Rayet B, Tan NS, Michalik L, Delaunay F, Wahli W, Laudet V (2006). Reciprocal regulation of brain and muscle Arnt-like protein 1 and peroxisome proliferator-activated receptor alpha defines a novel positive feedback loop in the rodent liver circadian clock. Mol Endocrinol.

[99] Bernal-Mizrachi C, Weng S, Feng C, Finck BN, Knutsen RH, Leone TC, Coleman T, Mecham RP, Kelly DP, Semenkovich CF (2003). Dexamethasone induction of hypertension and diabetes is PPAR-alpha dependent in LDL receptor-null mice. Nat Med.

[100] Wang C, Xu C-X, Krager SL, Bottum KM, Liao D-F, Tischkau SA (2011). Aryl hydrocarbon receptor deficiency enhances insulin sensitivity and reduces PPAR-α pathway activity in mice. Environ Health Perspect.

[101] Finck BN, Kelly DP (2002). Peroxisome proliferator-activated receptor alpha (PPARalpha) signaling in the gene regulatory control of energy metabolism in the normal and diseased heart. J Mol Cell Cardiol.

[102] Xu CX, Krager SL, Liao DF, Tischkau SA (2010). Disruption of CLOCK-BMAL1 transcriptional activity is responsible for aryl hydrocarbon receptor-mediated regulation of Period1 gene. Toxicol Sci.

[103] Baker NA, Karounos M, English V, Fang J, Wei Y, Stromberg A, Sunkara M, Morris AJ, Swanson HI, Cassis LA (2013). Coplanar polychlorinated biphenyls impair glucose homeostasis in lean C57BL/6 mice and mitigate beneficial effects of weight loss on glucose homeostasis in obese mice. Environ Health Perspect.

[104] Lee DH, Lee IK, Song K, Steffes M, Toscano W, Baker BA, Jacobs DR (2006). A strong dose-response relation between serum concentrations of persistent organic pollutants and diabetes: results from the National Health and Examination Survey 1999–2002. Diabetes Care.

[105] Baker NA, English V, Sunkara M, Morris AJ, Pearson KJ, Cassis LA (2013). Resveratrol protects against polychlorinated biphenyl-mediated impairment of glucose homeostasis in adipocytes. J Nutr Biochem.

[106] Dere E, Lo R, Celius T, Matthews J, Zacharewski TR (2011). Integration of genome-wide computation DRE search, AhR ChIP-chip and gene expression analyses of TCDD-elicited responses in the mouse liver. BMC Genomics.

[107] Diani-Moore S, Ram P, Li X, Mondal P, Youn DY, Sauve AA, Rifkind AB (2010). Identification of the aryl hydrocarbon receptor target gene TiPARP as a mediator of suppression of hepatic gluconeogenesis by 2,3,7,8-tetrachlorodibenzo-p-dioxin and of nicotinamide as a corrective agent for this effect. J Biol Chem.

[108] Enan E, Liu PC, Matsumura F (1992). 2,3,7,8-Tetrachlorodibenzo-p-dioxin causes reduction of glucose transporting activities in the plasma membranes of adipose tissue and pancreas from the guinea pig. J Biol Chem.

[109] Mueckler M, Kruse M, Strube M, Riggs AC, Chiu KC, Permutt MA (1994). A mutation in the Glut2 glucose transporter gene of a diabetic patient abolishes transport activity. J Biol Chem.

[110] Poland AP, Smith D, Metter G, Possick P (1971). A health survey of workers in a 2,4-D and 2,4,5-T plan with special attention to chloracne, porphyria cutanea tarda, and psychologic parameters. Arch Environ Health.

[111] Lakshman MR, Chirtel SJ, Chambers LL, Coutlakis PJ (1989). Effects of 2,3,7,8-tetrachlorodibenzo-p-dioxin on lipid synthesis and lipogenic enzymes in the rat. J Pharmacol Exp Ther.

[112] Lakshman MR, Campbell BS, Chirtel SJ, Ekarohita N (1988). Effects of 2,3,7,8-tetrachlorodibenzo-p-dioxin (TCDD) on de novo fatty acid and cholesterol synthesis in the rat. Lipids.

[113] Weber LW, Lebofsky M, Stahl BU, Gorski JR, Muzi G, Rozman K (1991). Reduced activities of key enzymes of gluconeogenesis as possible cause of acute toxicity of 2,3,7,8-tetrachlorodibenzo-p-dioxin (TCDD) in rats. Toxicology.

[114] Alexander DL, Ganem LG, Fernandez-Salguero P, Gonzalez F, Jefcoate CR (1998). Aryl-hydrocarbon receptor is an inhibitory regulator of lipid synthesis and of commitment to adipogenesis. J Cell Sci.

[115] Lee JH, Wada T, Febbraio M, He J, Matsubara T, Lee MJ, Gonzalez FJ, Xie W (2010). A novel role for the dioxin receptor in fatty acid metabolism and hepatic steatosis. Gastroenterology.

[116] Xu CX, Wang C, Zhang ZM, Jaeger CD, Krager SL, Bottum KM, Liu J, Liao DF, Tischkau SA (2015). Aryl hydrocarbon receptor deficiency protects mice from diet-induced adiposity and metabolic disorders through increased energy expenditure. Int J Obes.

[117] Rojas IY, Moyer BJ, Ringelberg CS, Tomlinson CR (2020). Reversal of obesity and liver steatosis in mice via inhibition of aryl hydrocarbon receptor and altered gene expression of CYP1B1, PPARα, SCD1, and osteopontin. Int J Obes.

[118] Li F, Jiang C, Larsen MC, Bushkofsky J, Krausz KW, Wang T, Jefcoate CR, Gonzalez FJ (2014). Lipidomics reveals a link between CYP1B1 and SCD1 in promoting obesity. J Proteome Res.

[119] Liu X, Huang T, Li L, Tang Y, Tian Y, Wang S, Fan C (2015). CYP1B1 deficiency ameliorates obesity and glucose intolerance induced by high fat diet in adult C57BL/6J mice. Am J Transl Res.

[120] Vasiliou V, Gonzalez FJ (2008). Role of CYP1B1 in glaucoma. Annu Rev Pharmacol Toxicol.

[121] Donovan EL, Pettine SM, Hickey MS, Hamilton KL, Miller BF (2013). Lipidomic analysis of human plasma reveals ether-linked lipids that are elevated in morbidly obese humans compared to lean. Diabetol Metab Syndr.

[122] Petersen MC, Vatner DF, Shulman GI (2017). Regulation of hepatic glucose metabolism in health and disease. Nat Rev Endocrinol.

[123] Zhang X, Yang S, Chen J, Su Z (2019). Unraveling the regulation of hepatic gluconeogenesis. Front Endocrinol.

[124] Korecka A, Dona A, Lahiri S, Tett AJ, Al-Asmakh M, Braniste V, D'Arienzo R, Abbaspour A, Reichardt N, Fujii-Kuriyama Y (2016). Bidirectional communication between the Aryl hydrocarbon receptor (AhR) and the microbiome tunes host metabolism. NPJ Biofilms Microbiomes.

[125] Lin C, Theodorides ML, McDaniel AH, Tordoff MG, Zhang Q, Li X, Bosak N, Bachmanov AA, Reed DR (2013). QTL analysis of dietary obesity in C57BL/6byj X 129P3/J F2 mice: diet- and sex-dependent effects. PLoS ONE.

[126] Mandl M, Depping R (2014). Hypoxia-inducible aryl hydrocarbon receptor nuclear translocator (ARNT) (HIF-1β): is it a rare exception?. Mol Med.

[127] Pillai R, Huypens P, Huang M, Schaefer S, Sheinin T, Wettig SD, Joseph JW (2011). Aryl hydrocarbon receptor nuclear translocator/hypoxia-inducible factor-1{beta} plays a critical role in maintaining glucose-stimulated anaplerosis and insulin release from pancreatic {beta}-cells. J Biol Chem.

[128] Abel J, Haarmann-Stemmann T (2010). An introduction to the molecular basics of aryl hydrocarbon receptor biology. Biol Chem.

[129] Zagórska A, Dulak J (2004). HIF-1: the knowns and unknowns of hypoxia sensing. Acta Biochim Pol.

[130] Yim SH, Shah Y, Tomita S, Morris HD, Gavrilova O, Lambert G, Ward JM, Gonzalez FJ (2006). Disruption of the Arnt gene in endothelial cells causes hepatic vascular defects and partial embryonic lethality in mice. Hepatology.

[131] Wang XL, Suzuki R, Lee K, Tran T, Gunton JE, Saha AK, Patti ME, Goldfine A, Ruderman NB, Gonzalez FJ (2009). Ablation of ARNT/HIF1beta in liver alters gluconeogenesis, lipogenic gene expression, and serum ketones. Cell Metab.

[132] Levisetti MG, Polonsky KS (2005). Diabetic pancreatic beta cells ARNT all they should be. Cell Metab.

[133] Czech M (2006). ARNT misbehavin' in diabetic beta cells. Nat Med.

[134] Stokes RA, Cheng K, Deters N, Lau SM, Hawthorne WJ, O'Connell PJ, Stolp J, Grey S, Loudovaris T, Kay TW (2013). Hypoxia-inducible factor-1α (HIF-1α) potentiates β-cell survival after islet transplantation of human and mouse islets. Cell Transpl.

[135] Lalwani A, Stokes R, Lau S, Gunton J (2014). Deletion of ARNT (Aryl hydrocarbon receptor nuclear translocator) in β-cells causes islet transplant failure with impaired β-cell function. PLoS ONE.

[136] Jensen MV, Joseph JW, Ronnebaum SM, Burgess SC, Sherry AD, Newgard CB (2008). Metabolic cycling in control of glucose-stimulated insulin secretion. Am J Physiol Endocrinol Metab.

[137] Hasan NM, Longacre MJ, Stoker SW, Boonsaen T, Jitrapakdee S, Kendrick MA, Wallace JC, MacDonald MJ (2008). Impaired anaplerosis and insulin secretion in insulinoma cells caused by small interfering RNA-mediated suppression of pyruvate carboxylase. J Biol Chem.

[138] Brownlee M (2001). Biochemistry and molecular cell biology of diabetic complications. Nature.

[139] Evans JL, Goldfine ID, Maddux BA, Grodsky GM (2002). Oxidative stress and stress-activated signaling pathways: a unifying hypothesis of type 2 diabetes. Endocr Rev.

[140] Ceriello A (2003). New insights on oxidative stress and diabetic complications may lead to a “causal” antioxidant therapy. Diabetes Care.

[141] Maiese K, Morhan SD, Chong ZZ (2007). Oxidative stress biology and cell injury during type 1 and type 2 diabetes mellitus. Curr Neurovasc Res.

[142] Matos MJ, Mura F, Vazquez-Rodriguez S, Borges F, Santana L, Uriarte E, Olea-Azar C (2015). Study of coumarin-resveratrol hybrids as potent antioxidant compounds. Molecules.

[143] Liu C, Wang Z, Song Y, Wu D, Zheng X, Li P, Jin J, Xu N, Li L (2015). Effects of berberine on amelioration of hyperglycemia and oxidative stress in high glucose and high fat diet-induced diabetic hamsters in vivo. Biomed Res Int.

[144] Anello M, Lupi R, Spampinato D, Piro S, Masini M, Boggi U, Del Prato S, Rabuazzo AM, Purrello F, Marchetti P (2005). Functional and morphological alterations of mitochondria in pancreatic beta cells from type 2 diabetic patients. Diabetologia.

[145] Segerstolpe Å, Palasantza A, Eliasson P, Andersson EM, Andréasson AC, Sun X, Picelli S, Sabirsh A, Clausen M, Bjursell MK (2016). Single-cell transcriptome profiling of human pancreatic islets in health and type 2 diabetes. Cell Metab.

[146] Cai L, Kang YJ (2001). Oxidative stress and diabetic cardiomyopathy: a brief review. Cardiovasc Toxicol.

[147] Tiwari BK, Pandey KB, Abidi AB, Rizvi SI (2013). Markers of oxidative stress during diabetes mellitus. J Biomark.

[148] Dietrich C (2016). Antioxidant functions of the aryl hydrocarbon receptor. Stem Cells Int.

[149] Lee WJ, Liu SH, Chiang CK, Lin SY, Liang KW, Chen CH, Tien HR, Chen PH, Wu JP, Tsai YC (2016). Aryl hydrocarbon receptor deficiency attenuates oxidative stress-related mesangial cell activation and macrophage infiltration and extracellular matrix accumulation in diabetic nephropathy. Antioxid Redox Signal.

[150] Xu L, Xing M, Xu X, Saadeldeen FS, Liu Z, Wei J, Kang W (2019). Alizarin increase glucose uptake through PI3K/Akt signaling and improve alloxan-induced diabetic mice. Future Med Chem.

[151] Sevanian A, Nordenbrand K, Kim E, Ernster L, Hochstein P (1990). Microsomal lipid peroxidation: the role of NADPH–cytochrome P450 reductase and cytochrome P450. Free Radic Biol Med.

[152] Vianna CR, Huntgeburth M, Coppari R, Choi CS, Lin J, Krauss S, Barbatelli G, Tzameli I, Kim Y-B, Cinti S (2006). Hypomorphic mutation of PGC-1beta causes mitochondrial dysfunction and liver insulin resistance. Cell Metab.

[153] American Diabetes Association (2009). Diagnosis and classification of diabetes mellitus. Diabetes Care.

[154] Kanneganti TD, Dixit VD (2012). Immunological complications of obesity. Nat Immunol.

[155] Shu CJ, Benoist C, Mathis D (2012). The immune system's involvement in obesity-driven type 2 diabetes. Semin Immunol.

[156] Ndisang JF, Rastogi S, Vannacci A (2014). Immune and inflammatory processes in obesity, insulin resistance, diabetes, and related cardiometabolic complications. J Immunol Res.

[157] Zhao RX, Li WJ, Lu YR, Qin J, Wu CL, Tian M, He TY, Yi SN, Tang DQ, Sun L (2014). Increased peripheral proinflammatory T helper subsets contribute to cardiovascular complications in diabetic patients. Mediat Inflamm.

[158] Lumeng CN, Maillard I, Saltiel AR (2009). T-ing up inflammation in fat. Nat Med.

[159] Gutiérrez-Vázquez C, Quintana FJ (2018). Regulation of the immune response by the aryl hydrocarbon receptor. Immunity.

[160] Baker NA, Shoemaker R, English V, Larian N, Sunkara M, Morris AJ, Walker M, Yiannikouris F, Cassis LA (2015). Effects of adipocyte aryl hydrocarbon receptor deficiency on pcb-induced disruption of glucose homeostasis in lean and obese mice. Environ Health Perspect.

[161] Zhao RX, He Q, Sha S, Song J, Qin J, Liu P, Sun YJ, Sun L, Hou XG, Chen L (2020). Increased AHR transcripts correlate with pro-inflammatory T-helper lymphocytes polarization in both metabolically healthy obesity and type 2 diabetic patients. Front Immunol.

[162] Unanue E, Byersdorfer C, Carrero J, Levisetti M, Lovitch S, Pu Z, Suri A (2005). Antigen presentation: lysoyme, autoimmune diabetes, and Listeria–what do they have in common?. Immunol Res.

[163] Kimura A, Naka T, Nakahama T, Chinen I, Masuda K, Nohara K, Fujii-Kuriyama Y, Kishimoto T (2009). Aryl hydrocarbon receptor in combination with Stat1 regulates LPS-induced inflammatory responses. J Exp Med.

[164] Kim MJ, Pelloux V, Guyot E, Tordjman J, Bui LC, Chevallier A, Forest C, Benelli C, Clement K, Barouki R (2012). Inflammatory pathway genes belong to major targets of persistent organic pollutants in adipose cells. Environ Health Perspect.

[165] Kerkvliet NI, Steppan LB, Vorachek W, Oda S, Farrer D, Wong CP, Pham D, Mourich DV (2009). Activation of aryl hydrocarbon receptor by TCDD prevents diabetes in NOD mice and increases Foxp3+ T cells in pancreatic lymph nodes. Immunotherapy.

[166] Reed MJ, Meszaros K, Entes LJ, Claypool MD, Pinkett JG, Gadbois TM, Reaven GM (2000). A new rat model of type 2 diabetes: the fat-fed, streptozotocin-treated rat. Metabolism.

[167] Sawant SP, Dnyanmote AV, Shankar K, Limaye PB, Latendresse JR, Mehendale HM (2004). Potentiation of carbon tetrachloride hepatotoxicity and lethality in type 2 diabetic rats. J Pharmacol Exp Ther.

[168] Fried KW, Guo GL, Esterly N, Kong B, Rozman KK (2010). 2,3,7,8-tetrachlorodibenzo-p-dioxin (TCDD) reverses hyperglycemia in a type II diabetes mellitus rat model by a mechanism unrelated to PPAR gamma. Drug Chem Toxicol.

[169] Leijs MM, Koppe JG, Vulsma T, Olie K, van Aalderen WMC, de Voogt P, Legler J, Ten Tusscher GW (2017). Alterations in the programming of energy metabolism in adolescents with background exposure to dioxins, dl-PCBs and PBDEs. PLoS ONE.

[170] Warner M, Rauch S, Brambilla P, Signorini S, Mocarelli P, Eskenazi B (2020). Prenatal dioxin exposure and glucose metabolism in the Seveso Second Generation study. Environ Int.

[171] Lin Y-H, Luck H, Khan S, Schneeberger PHH, Tsai S, Clemente-Casares X, Lei H, Leu Y-L, Chan YT, Chen H-Y (2019). Aryl hydrocarbon receptor agonist indigo protects against obesity-related insulin resistance through modulation of intestinal and metabolic tissue immunity. Int J Obes.

